# Vibrational spectra of formic acid and its dimer: II. Towards identifying structural motifs in atmospheric organic clustering

**DOI:** 10.1039/d6cp01819j

**Published:** 2026-07-22

**Authors:** Dennis F. Dinu, Vincent Enders, Julius Stolze, Lukas Meinschad, Jonas Schlagin, Klaus R. Liedl, Guntram Rauhut, Thomas Loerting, Hinrich Grothe, Maren Podewitz, Dominik Stolzenburg

**Affiliations:** a Institute of Materials Chemistry, TU Wien Getreidemarkt 9 1060 Wien Austria dominik.stolzenburg@tuwien.ac.at; b Department of General, Inorganic and Theoretical Chemistry, University of Innsbruck Innrain 80 6020 Innsbruck Austria; c Department of Physical Chemistry, University of Innsbruck Innrain 52 6020 Innsbruck Austria; d Institute of Theoretical Chemistry, University of Stuttgart Pfaffenwaldring 55 70569 Stuttgart Germany

## Abstract

This work constitutes part II of a series on vibrational spectroscopy of formic acid, focusing on the molecular interactions of its carboxyl group during dimerization. Such interactions drive the earliest steps of clustering in organic-mediated atmospheric new particle formation (NPF), and are difficult to access with the mass spectrometric methods typically used to study NPF. While theoretical thermochemistry provides binding free energies for various cluster conformers, these conformers have not been experimentally probed to date, and it is unclear which conformers ultimately participate in atmospheric organic clustering. Vibrational spectroscopy using matrix-isolation provides a route to experimentally investigate the conformers of small clusters. To identify simple conformers in the clustering of the carboxyl group, we use the model system of the formic acid monomer and dimer in cryogenic matrices in combination with *ab initio* calculations. We find that: (1) efficient harmonic frequency calculations, even at a low level of density functional theory, reproduce both the direction and magnitude of the experimentally observed dimer-formation frequency shifts across different conformers. (2) Matrix effects effectively cancel out when investigating such shifts. (3) The most reliable region to study these shifts is the *ν*C=O and *ν*C–O regions, supported secondarily by the broader and less intense *ν*OH and *δ*_oop_COH regions. (4) Under cryogenic matrix-isolation conditions, thermodynamically less favorable conformers appear with relatively high abundance, suggesting that the matrix serves as a kinetic trap. Altogether, we can capture structural information about short-lived conformers and thus demonstrate their experimental existence. This supports the common practice of including less favorable conformers in *ab initio* computational nucleation models, as they are considered intermediates in atmospheric organic clustering. Our insights from this model system of formic acid pave the way for future studies of organic clustering analyzed in cryogenic matrices to investigate the earliest steps of sub-nanometer NPF.

## Abstract 2

Matrix-isolation FTIR spectroscopy and *ab initio* calculations enable identification of formic acid dimer conformers and structural changes during carboxyl group clustering, providing molecular insights into early atmospheric cluster chemistry.

## Graphical abstract

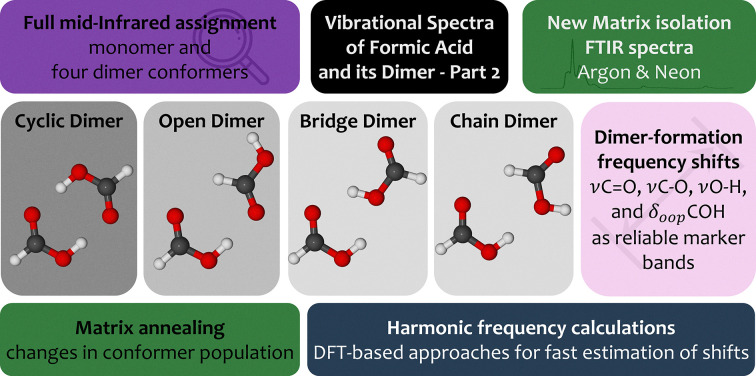

## 1 Introduction

**Fig. 1:**
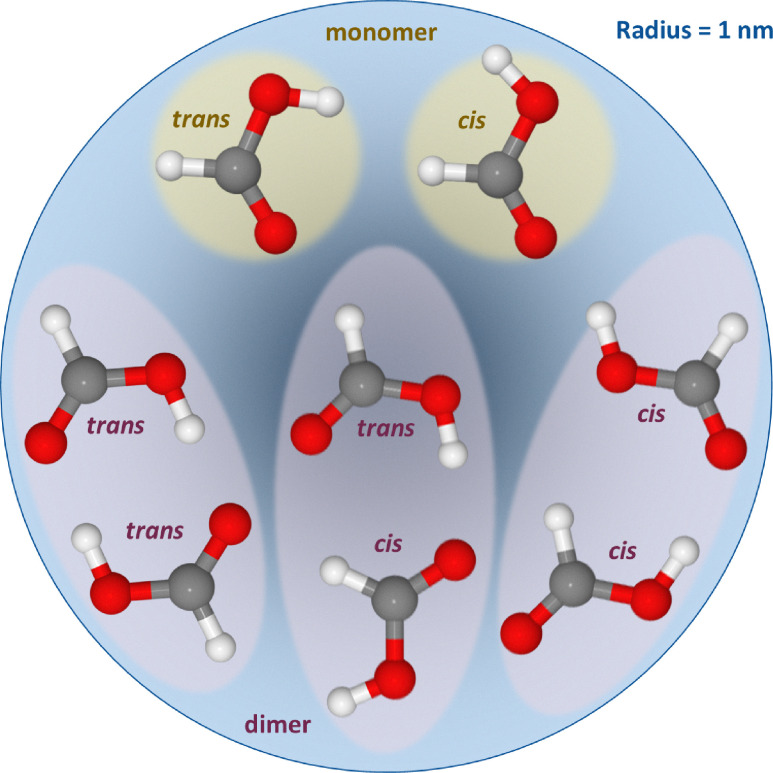
Schematic depiction of how many formic acid molecules can be accommodated within a hypothetical two-dimensional nanoparticle (blue) with a radius of 1 nm. Formic acid is present as *trans* and *cis* monomers (yellow), which can interact to generate different dimers (purple).

In part I, we established a spectroscopic benchmark for formic acid and its cyclic dimer using anharmonic vibrational treatments. Here, in part II, we build on this foundation to investigate the role of conformational diversity in atmospheric new particle formation (NPF). This process, in which low-volatility gases form molecular clusters (*cf.*Fig. 1) that subsequently grow into larger liquid- or solid-phase aerosol particles,^1^ is a global phenomenon^2,3^ that influences climate and air quality.^4,5^ NPF involves the chemical reactions that transform volatile gases into less-volatile, and hence condensable, species; the initial clustering of a few molecules, often called nucleation; and the phase-transition dynamics of the growing particles. Depending on the environment, different species contribute to the different stages of NPF.^6–13^

Molecules involved in NPF are commonly investigated using mass spectrometry,^14^ often combined with soft chemical ionization at atmospheric pressure.^15,16^ However, while increasing the mass resolution of atmospheric-pressure-interface mass spectrometers has enabled the identification of the atomic constituents of molecules participating in NPF,^17,18^ this technique lacks structural information. Tandem mass spectrometry (MS–MS) techniques could, in principle, elucidate molecular arrangements in newly formed atmospheric clusters; however, they have not been widely applied to NPF studies.^19,20^ Infrared (IR) spectroscopy, in contrast, provides insights into molecular structure. It has been widely used for bulk aerosol chemical characterization^21–24^ but it can also be used to study cluster arrangements and therefore it is well suited to also study the initial steps of NPF.^25^

An instructive application was presented by Gadermann *et al.* (2007), who used Fourier-transform infrared (FTIR) spectroscopy to study acetic and formic acid aerosols generated in a collisional cooling cell at 78 K.^26^ Their setup enabled investigation of aerosol particles as small as 1 nm. This information is valuable, particularly because formic acid contributes significantly to the acidity of precipitation and cloud water.^27,28^ In NPF, it was long thought that inorganic species dominated the nucleation steps,^1^ but in recent years it was found that organics can also nucleate in the absence of sulfuric acid.^8^ However, understanding organic clustering is a significant challenge to our understanding of NPF, given the myriad organic compounds in the atmosphere.^29^ Only very specific molecular properties could allow the formation of stable clusters.^30^ In organic-mediated NPF, highly oxygenated molecules, so-called HOMs, often originating from the oxidation of large volatile organic compounds, are thought to be key components.^30,31^ While formic acid is not a key component of atmospheric clustering by itself, it is speculated to contribute to the stabilization of the initial clustering of other molecules, such as sulfuric acid or HOMs.^32,33^ As carboxylic acids might generally take a key role in organic-mediated NPF,^34,35^ studying one of the simplest atmospheric organic acids, formic acid, could generally pave the way towards understanding the atmospheric organic clustering of more complex molecules, as already indicated by Gadermann *et al.* (2007).^26^

This understanding is not only crucial from a molecular-structure perspective but also from a practical one. For example, the computational atmospheric cluster database of sulfuric acid, bases, organics, and water introduced by Elm in 2019^36^ already contains more than 600 clusters. An issue is the immense number of conformations that arise from molecular clustering, which is itself a considerable computational challenge, as recently reviewed by Elm *et al.*^37^ To disentangle this vast conformational space, Kubečka *et al.*^38^ proposed a combination of computational tools, with density functional theory calculations serving as the key method for obtaining thermodynamically reasonable sets of conformers. From these spaces, statistical thermochemistry and classical nucleation theory can be used to predict nucleation rates.^39^ However, the cluster formation energies associated with initial monomer interactions are practically inaccessible to typical experiments used in atmospheric chemistry.^37^

**Fig. 2:**
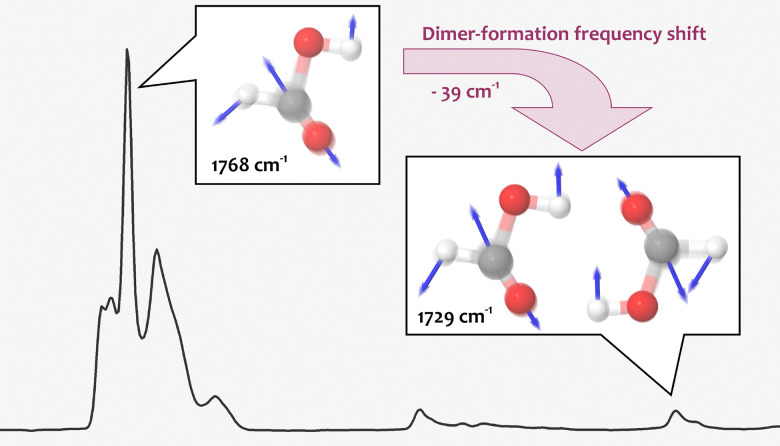
Matrix-isolation Fourier-transform Infrared (MI-FTIR) spectrum of formic acid isolated in argon, showing the *ν*C=O stretching vibration. The vibration occurring in a cluster of two formic acid molecules, *i.e.*, the dimer (right insert), is shifted towards lower vibrational frequencies compared to a single formic acid molecule, *i.e.* the monomer (left insert).

Considering two hydrogen-bonded formic acid molecules, their size is significantly below 1 nm, as depicted in Fig. 1. Only the addition of further formic acid molecules leads to the growth of clusters approaching the 1 nm particle regime. IR spectroscopy is well suited to study such molecular aggregation because the associated conformational changes and hydrogen-bonding interactions induce subtle dimer-formation frequency shifts (*cf.*Fig. 2), an effect well known since the early work of Badger and Bauer.^40^ These shifts can be linked to structural changes, making IR spectroscopy a powerful tool for investigating molecular arrangements during clustering. A promising technique for observing dimer-formation frequency shifts is to “freeze” molecular clusters in a cryogenic matrix and probe the infrared absorption of the nanolayer. This approach, typically denoted as matrix-isolation Fourier-transform infrared (MI-FTIR) spectroscopy, quenches molecular rotation and enables a straightforward comparison of vibrations between monomers and clusters. Also, in MI-FTIR, species that are less stable in the gas phase can be observed. Pioneering work in this field was done by Rozenberg *et al.* (2009, 2014),^41,42^ who investigated the frequency shifts associated with the formation of sulfuric acid–water and sulfuric acid–base clusters. Ultimately, these shifts can be translated into structural insights that complement standard methodologies for studying NPF and advance our understanding of atmospheric nucleation mechanisms. However, after the pioneering work by Rosenberg and co-workers, MI-FTIR has not been directly used to study clustering involving organics, where intermolecular binding is likely dominated by carboxyl groups. In that sense, formic acid, as the simplest organic acid, can serve as a model system to outline how organic clustering relevant to atmospheric NPF can be best studied with this technique.

If we study how the vibration changes upon cluster formation, we can define cluster-formation frequency shifts. If we want to compare a vibration in a small molecular system (*e.g.*, the monomer) to a vibration in a larger one (*e.g.*, the dimer), this leads to the dimer-formation frequency shift. From a chemical perspective, one typically compares “similar” vibrations, such as the *ν*C=O vibration in the monomer (*cf.*Fig. 2 left) with its “counterpart” in the dimer (*cf.*Fig. 2 right). While this approach is intuitively appealing and informative, it should be noted that it entails projections between different vibrational manifolds. The difference between the spectrum of a monomer and a dimer can be crudely separated into two extremes: (A) the dimer has generally more vibrational states of similar character. For example, while the *ν*C=O vibration is observed as one band in the spectrum of the monomer, it can contribute to multiple bands in the spectrum of the dimer. (B) The dimer has more degrees of freedom than the monomer; hence, the conformational space is larger. For example, there is only the “free” *ν*C=O vibration in the monomer, both in the *trans*- and *cis*-conformer. For the dimer, there can be “bridged” and “free” *ν*C=O vibrations, depending on whether the dimer is in a cyclic or open conformer. Ultimately, a combination of (A) and (B) results in distinct dimer-formation frequency shifts.

For NPF, the main challenge is to understand these shifts without relying too heavily on expensive *ab initio* calculations. As noted above, atmospheric cluster databases require immense numbers of clusters and conformers for reliable thermochemistry predictions. Calculating anharmonic spectra for all possible clusters and conformers is not feasible. Harmonic frequency calculations offer a practical alternative. Chang *et al.* have shown that formic acid dimerization frequency shifts from the harmonic approximation are comparable to experimental observations already in 1987.^43^ Today, the fortunate error compensation of such harmonic calculations is well understood and benchmarked, *e.g.* for the methanol dimer.^44,45^ This was also prominently demonstrated for hydrogen-bonded OH stretching fundamentals of water in various monohydrates, during the 2021 HyDRA blind challenge.^46^ However, whether this error compensation in the harmonic approximation is reliable for analytical applications of FTIR in organic-mediated NPF studies remains to be validated.

The overall task here is to identify – with the model system of formic acid – the vibrational modes which convey the most robust information on organic clustering using MI-FTIR spectroscopy, considering: (1) the resolving power of the approach, *i.e.*, the magnitude of the band shifts, intensities of the peaks, experimental uncertainties due to matrix-effects and overlap of modes; (2) theoretical accuracy (DFT level of theory, harmonic or anharmonic approaches) of predicting band shifts such that different conformers can be identified. Here, we aim to determine what can and cannot be achieved by combining theoretical calculations with MI-FTIR experiments optimized to observe cluster formation involving organic acids, thereby enabling us to expand our investigations to more complex organic molecules directly relevant to atmospheric NPF in future studies.

## 2 Methodology

### 2.1 Matrix annealing experiments

We use the same experimental methodology as described in part I of our study,^47^ where we primarily observed the cyclic formic acid dimer *via* different guest–host mixing ratios. Here, we perform annealing experiments to leverage the possibility of different dimer conformer formation in the matrix. For these experiments, approximately 3 ml of high-purity liquid HCOOH (98%, Sigma-Aldrich, 1.00264) was transferred *via* sterile syringe into a glass flask mounted to the mixing chamber and subsequently degassed *via* freeze–pump–thaw cycles. Mixtures with host gases were prepared in the mixing chamber (at 298 K and 10^−5^ mbar). We produced guest-to-host dilutions of 1 : 250 in both argon and neon. The matrix was deposited at 5.0 K using a mass flow controller to achieve slow deposition and create a thick layer, which is necessary for the subsequent annealing experiment. Annealing was performed by stepwise heating of the matrix sample using a PID controller. For argon, the matrix began to evaporate at approximately 30 K; for neon, at approximately 8 K. The spectra were measured in the 8000–500 cm^−1^ range using 32 scans; background spectra at 5.0 K were subtracted from the sample spectra.

### 2.2 Conformational sampling, thermochemistry, harmonic calculations

We employ a computational methodology that includes conformational sampling, thermochemistry, and harmonic frequency calculations for various conformers. First, we explored the conformational space using an approach that was motivated by the “Jammy Key” framework by Kubecka *et al.*^38^ Conformational ensembles of the formic acid dimer were generated using the conformer–rotamer ensemble sampling tool (CREST)^48^ in its non-covalent interaction mode. As the starting geometry, we chose the cyclic dimer of *trans*-formic acid. With the default CREST settings, only a limited number of conformers are found, as the cyclic formic acid dimer is exceptionally stable. To obtain a more comprehensive conformational space, we therefore relaxed the selection criteria by increasing the metadynamics simulation time by a factor of five (–mdlen × 5.0) and retaining conformers within an energy window of 300 kcal mol^−1^ (–ewin 300). In addition, the sampling employed a conformer-pair energy threshold of 0.005 kcal mol^−1^ (–ethr 0.005). All computations were performed with the GFN2-xTB method.^49^

The CREST run yielded 127 structures. Although this includes a broad range of conformers, most were unsuitable because the two subunits in the dimer were too far apart. We therefore retained only those conformers whose intermolecular distances permitted hydrogen bonding or weaker non-covalent interactions, yielding a subset of 17 lowest-energy structures. These were subsequently subjected to geometry optimization by density functional theory using the *ω*B97X-3c method^50^ using ORCA version 6.0.^51^ The *ω*B97X-3c method employs a specially optimized polarized valence double-*ζ* basis set together with dispersion correction. Owing to its composite design, basis set superposition errors (BSSE) are reduced and residual effects are effectively absorbed by the dispersion correction damping scheme, such that no explicit BSSE correction was applied.

Thermochemical properties at standard conditions (298 K, 1 atm) were evaluated from the harmonic frequencies of the optimized geometries of these 17 conformers, employing the quasi rigid-rotor/harmonic-oscillator model as implemented by default in ORCA. No frequency scaling was applied. The conformers were then ordered according to their Gibbs free energy of formation, which is computed as the difference Δ*G*_f_ = *G*[dimer] − 2·*G*[monomer].

We calculate the harmonic vibrational frequencies using different levels of theory for the lowest-energy conformer, as shown in the SI. We compare these harmonic vibrational frequency calculations to results from part I of our series,^47^ where we focused on anharmonic calculations using vibrational configuration interaction theory (VCI) and vibrational perturbation theory (VPT2) based on a newly constructed *ab initio* potential energy surface at CCSD(T)-F12 level of theory. For the final study of all conformers, we rely on harmonic vibrational frequencies based on the *ω*B97X-3c level of theory. Using the analytical Hessians, we applied the nomodeco toolkit to perform normal-mode decomposition and to characterize the vibrational modes in terms of internal coordinates.^52^ For IC set generation in hydrogen-bonded clusters, all hydrogen-bond coordinates, together with donor–acceptor bonds and angles, were included (–comb 2).^53^

## 3 Results and discussion

**Fig. 3:**
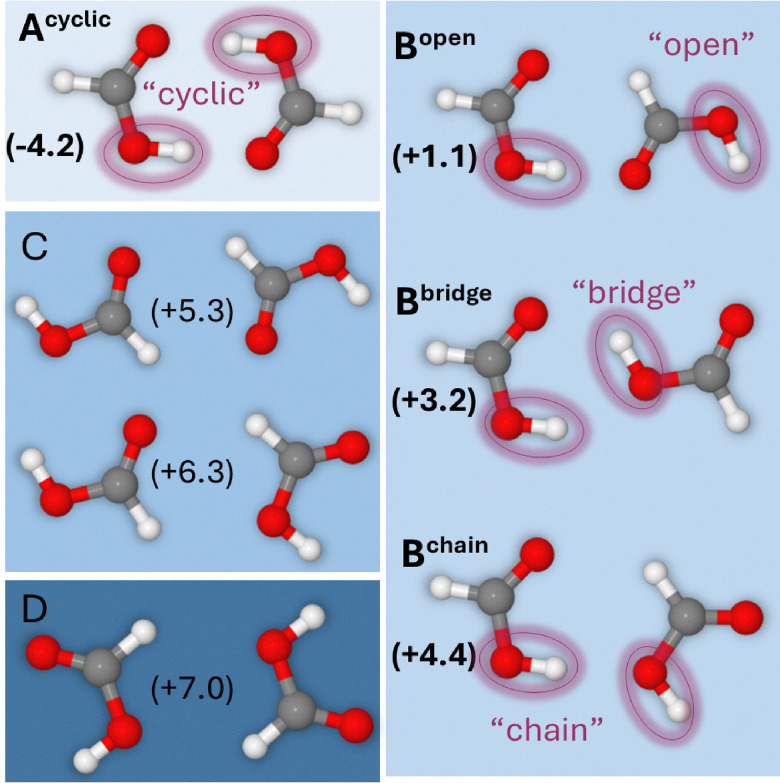
Conformers of dimers formed by *trans*-formic acid, as determined from CREST calculations and refined using density functional theory with the *ω*B97X-3c method. The Gibbs free energy of dimer formation under standard conditions is presented in kcal mol^−1^ within brackets.

In part I of this series,^47^ we focused on the accuracy of computational approaches for the assignment of all observed features in the MI-FTIR spectra of the *trans*-formic acid monomer (hereafter denoted as **M**) and its cyclic dimer with *C*_2h_ symmetry (hereafter denoted as **A**^cyc^, *cf.*Fig. 3). We have shown that anharmonic calculations can achieve spectroscopic accuracy in MI-FTIR experiments, whereas harmonic calculations cannot. In the following, we complete this investigation with a distinct focus on dimer-formation frequency shifts. We define the dimer-formation frequency shift as:*Δ*^*ν*OH^_Gas_ = *ν̃*^*ν*OH^_Gas_(dimer) − *ν̃*^*ν*OH^_Gas_(monomer).

Using the absolute wavenumbers *ν̃* from a given data set, such as obtained by gas phase IR spectroscopy, we obtain the dimer-formation frequency shift *Δ*_Gas_. This can result in a negative shift towards lower wavenumbers (redshift), or a positive shift towards higher wavenumbers (blueshift). In the given example, we compare the *ν*OH vibration in the monomer *ν̃*^*ν*OH^(monomer) with its “counterpart” in the dimer *ν̃*^*ν*OH^(dimer). Ultimately, we obtain the respective dimer-formation frequency shift of the *Δ*^*ν*OH^_Gas_ for a given vibration and dataset.

### 3.1 Computed dimer-formation frequency shifts of the cyclic dimer

Comparing the absolute frequencies listed in Table 1, we observe that anharmonic treatments (VCI and VPT2) yield frequencies in close agreement with gas phase experiment as shown in part I of this series already.^47^ The harmonic treatments systematically overestimate the vibrational frequencies, both using “high level” of wave function theory such as CCSD(T)-F12/cc-PVTZ-F12 (abbreviated as CC) and “low level” of density functional theory, here using the composite *ω*B97X-3c approach (abbreviated as DFT). As can be seen in the SI, the harmonic calculations generally overestimate the absolute frequency for any chosen functional or electronic structure theory. However, this overestimation affects both the monomer and the dimer, so the resulting errors cancel when considering frequency shifts. As a consequence, the calculated harmonic dimer-formation frequency shifts (*Δ*_CC_ for “high level” and *Δ*_DFT_ for “low level”) are therefore often in reasonable agreement with experimentally observed dimer-formation frequency shifts in the gas phase (*Δ*_Gas_) and anharmonic dimer-formation frequency shifts (*Δ*_VCI_ and *Δ*_VPT2_). We observe some systematic patterns in the accuracy of the predicted shifts and peculiarity related to specific functional groups (OH, CH, OCO) of the system, as discussed below.

**Table 1:** Vibrational frequencies of the *trans*-formic acid monomer (**M**) and its cyclic hydrogen-bonded dimer (**A**^cyc^), together with the corresponding frequency shifts upon dimer formation, as determined by IR spectroscopic measurements and theoretical calculations

Label		Experiment^a^							Anharmonic calculation^b^			Harmonic calculation^c^			
		Argon	*Δ*_Ar_	Neon	*Δ*_Ne_	Gas	*Δ*_Gas_	VCI	*Δ*_VCI_	VPT2	*Δ*_VPT2_	CC	*Δ*_CC_	DFT	*Δ*_DFT_
**M**	*ν*OH	3550		3570		3571		3572		3568		3761		3862	
**A** ^cyc^		3072	−478	3078	−492	3084	−487	3031	−541	3095	−473	3310	−451	3299	−563
**M**	*ν*C–H	2955		2939		2942		2940		2943		3091		3165	
**A** ^cyc^						2939	−3	2955	15	2907	−36	3099	8	3171	6
**M**	*ν*C=O	1768		1774		1777		1778		1779		1814		1897	
**A** ^cyc^		1729	−39	1738	−36	1746	−31	1742	−36	1741	−38	1782	−32	1856	−42
**M**	*δ*_ip_CH	1381		1380		1379		1379		1378		1409		1428	
**A** ^cyc^						1407	28	1404	25	1404	26	1455	46	1465	38
**M**	*δ*_ip_COH	1306		1305		1306		1311		1302		1318		1336	
**A** ^cyc^		1371	65	1373	67	1372	66	1370	60	1362	60	1406	89	1437	102
**M**	*ν*C–O	1104		1103		1105		1106		1106		1138		1184	
**A** ^cyc^		1227	123	1228	125	1230	125	1230	124	1229	123	1258	120	1314	130
**M**	*δ*_oop_CH	1038		1036		1034		1033		1033		1055		1076	
**A** ^cyc^		1070	32	1066	30	1069	36	1056	23	1058	25	1098	43	1118	42
**M**	*δ*_oop_COH	636		638		641		642		636		672		681	
**A** ^cyc^		940	304	943	305	944	303	936	294	944	308	983	311	1018	338
**M**	*δ*_ip_OCO	629		626		626		626		627		632		650	
**A** ^cyc^		712	83	707	80	708	82	708	82	705	78	714	82	740	90

a Matrix isolation FTIR spectra obtained in argon and neon matrices in the present study (with the exception of the *ν*CH band in argon at 2947 cm^−1^ adopted from Ito^54^ and the *δ*_ip_CH band in argon at 1445 cm^−1^ taken from Gantenberg^55^) are compared with gas phase reference data as compiled by Nejad.^56^

b Anharmonic vibrational calculations in this work were carried out using vibrational configuration interaction (VCI) and, alternatively, second-order vibrational perturbation theory (VPT2).

c Harmonic frequency calculations were performed at the CCSD(T)-F12/cc-pVTZ-F12 level of theory (“high-level”),^57,58^ and at computationally economical harmonic calculations at the *ω*B97X-3c level of theory (“low-level”).^50^ No empirical scaling factors were applied to the computed vibrational frequencies. Results from other functionals and electronic structure approaches are listed in the SI.

#### 3.1.1 OH related vibrations

The *Δ*^*ν*OH^ shift is the largest observed in the whole dataset (*Δ*^*ν*OH^_Gas_ = −487 cm^−1^). The harmonic calculation overestimates (*Δ*^*ν*OH^_DFT_ = −563 cm^−1^) or underestimates (*Δ*^*ν*OH^_CC_ = −451 cm^−1^) the shift, depending on the chosen electronic structure theory. Anharmonic treatments based on the same potential energy surface (*cf.* part I of our study^47^) also lead to both over- and underestimation (*Δ*^*ν*OH^_VCI_ = −541 cm^−1^ and *Δ*^*ν*OH^_VPT2_ = −473 cm^−1^). This pronounced sensitivity underscores the strongly anharmonic character of the hydrogen-bonded *ν*OH stretching mode, which depends critically on both the quality of the underlying potential energy surface and the specific treatment of anharmonicity. Consequently, computed *Δ*^*ν*OH^ shifts are prone to various sources of errors that may or may not compensate and, thus, must be interpreted with particular caution.

For the *Δ*^*δ*_ip_COH^ shift, the gas phase reference 
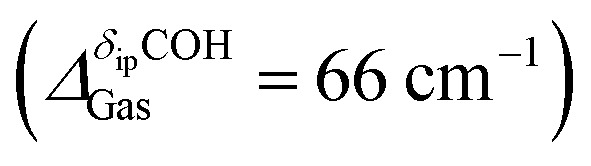
 is only qualitatively predicted by harmonic calculations 
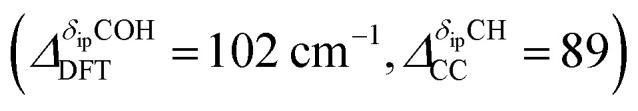
. Anharmonic calculations enable a more accurate prediction of the shift 
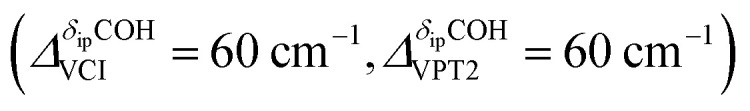
. The source of this discrepancy is a resonance phenomenon involving the *trans*-formic acid monomer **M**. VCI anharmonic calculations show that this resonance, which mixes the fundamental of *δ*_ip_COH with the first overtone of *δ*_oop_OH, yields two energy levels (1310.5 cm^−1^ and 1230.6 cm^−1^).^47^ As a result, two distinct frequency shifts can be defined 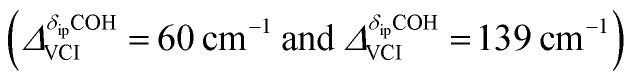, depending on whether the lower- or higher-frequency member of the resonant pair is used as reference. Within the harmonic approximation, this resonance is not reproduced, and the predicted shift corresponds almost exactly to the midpoint between these two limiting values. A simple and practical way to flag such problematic cases using only harmonic frequencies is to search for possible resonance pairs. In the present example, twice the harmonic frequency of *δ*_oop_OH is 1332 cm^−1^, which is very close to the harmonic frequency of *δ*_ip_COH at 1336 cm^−1^. This suggests that this specific combination of modes is likely to be problematic.

The *Δ*^*δ*_oop_COH^ shift is very pronounced in the gas phase reference 
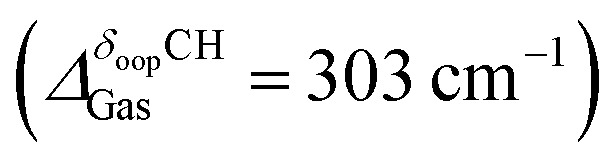
 and well reproduced by all theoretical approaches 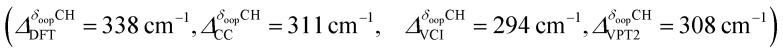. Due to this straightforward theoretical prediction, the *Δ*^*δ*_oop_COH^ shift may be the most useful marker for studying the dimer formation, considering the OH-related vibrations.

#### 3.1.2 CH related vibrations

The *Δ*^*ν*CH^ shift we obtained from the available gas phase references (*Δ*^*ν*CH^_Gas_ = −3 cm^−1^) is suspiciously inconsistent with any of the calculated shifts, which vary in sign and magnitude, depending on the chosen method (*Δ*^*ν*CH^_VCI_ = 15 cm^−1^, *Δ*^*ν*CH^_VPT2_ = −36 cm^−1^, *Δ*^*ν*CH^_CC_ = 8 cm^−1^, and *Δ*^*ν*CH^_DFT_ = 8 cm^−1^). In their gas phase experiments, Freytes *et al.*^59^ and Georges *et al.*^60^ mention the difficulty in distinguishing the monomer and dimer. In the latter work, a less used assignment for the dimer *ν*CH vibration at 2957 cm^−1^ is mentioned, which would better fit (*Δ*^*ν*CH^_Gas_ = 15 cm^−1^) to our calculated shifts. Although this comparison shows the potential of using dimer-formation frequency shifts as an indicator of wrong assignments, it should become clear that the *Δ*^*ν*CH^ shift in general is a poor choice to distinguish dimer and monomer. For the *Δ*^*δ*_ip_CH^ shift, the gas phase reference 
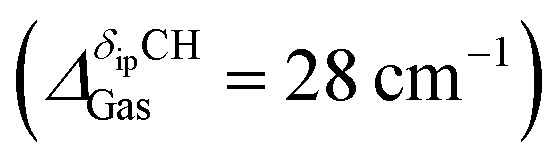
 is qualitatively predicted by harmonic calculations 
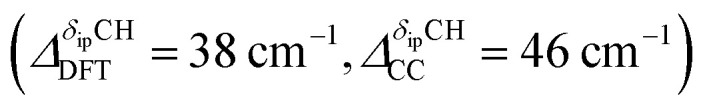
, but it needs anharmonic calculations for quantitative prediction 
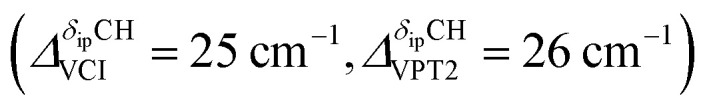
. For the *Δ*^*δ*_oop_CH^ shift, the gas phase reference 
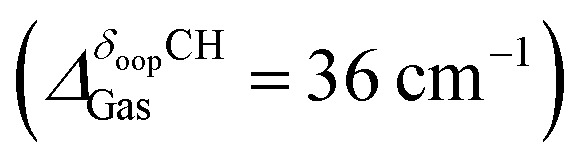
 is reasonably well predicted by harmonic calculations 
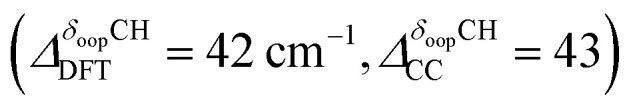
 and anharmonic calculations 
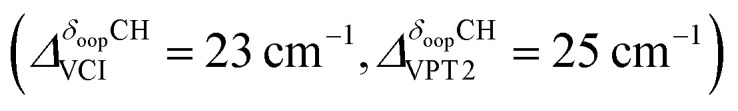
. Ultimately, the predictions for the *Δ*^*δ*_ip_CH^ and *Δ*^*δ*_oop_CH^ shifts are less reliable and likely not as well suited to study dimer formation.

#### 3.1.3 OCO related vibrations

The *Δ*^*ν*C=O^ shift from the gas phase experiment (*Δ*^*ν*C=O^_Gas_ = −31 cm^−1^) agrees well with the predictions by either theoretical approach. The “high level” harmonic calculations agree better with experiment (*Δ*^*ν*C=O^_DFT_ = −42 cm^−1^, *Δ*^*ν*C=O^_CC_ = −32 cm^−1^). The anharmonic calculations also slightly overestimate the magnitude of the shift (*Δ*^*ν*C=O^_VCI_ = −36 cm^−1^, *Δ*^*ν*C=O^_VPT2_ = −38 cm^−1^). Overall, the changes in the carbonyl bonding environment upon dimerization are described adequately by both anharmonic and harmonic calculations, making the *Δ*^*ν*C=O^ shift a good candidate for studying the dimer formations. The *Δ*^*ν*C–O^ shift from the gas phase experiment (*Δ*^*ν*C–O^_Gas_ = 123 cm^−1^) agrees perfectly with the predictions by either theoretical approaches (*Δ*^*ν*C–O^_DFT_ = 130 cm^−1^, *Δ*^*ν*C–O^_CC_ = 120 cm^−1^, *Δ*^*ν*C–O^_VCI_ = 124 cm^−1^, *Δ*^*ν*C–O^_VPT2_ = 123 cm^−1^). This makes the *Δ*^*ν*C–O^ shift another good candidate for studying the dimer formation. The same holds for the *Δ*^*δ*_ip_OCO^ shift. It is relatively large in the gas phase reference 
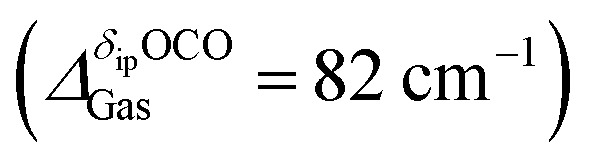
 and well reproduced by all theoretical approaches 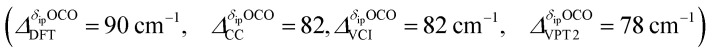.

### 3.2 Matrix effects on dimer-formation frequency shifts of the cyclic dimer

In contrast to gas-phase reference data, which are obtained from a broad range of highly specialized experimental techniques, MI-FTIR spectroscopy constitutes a practical methodology for acquiring comparable information on vibrational structure with substantially reduced experimental effort (*cf.* part I^47^). However, interactions with the rare-gas host matrix give rise to non-systematic, matrix-induced frequency shifts. However, as shown in Table 1, the dimer-formation frequency shifts determined by MI-FTIR spectroscopy in argon and neon matrices (*Δ*_Ar_, *Δ*_Ne_) reproduce the trends of the corresponding gas phase reference values (*Δ*_Gas_). Matrix effects therefore affect the experimentally observed dimer-formation frequency shifts only marginally, to an extent that is in fact smaller than the theoretical uncertainties discussed in Section 3.1. Nonetheless, the dimer-formation frequency shifts (*Δ*_Ar_, *Δ*_Ne_) extracted from MI-FTIR spectra are “apparent” shifts. These values comprise not only the intrinsic spectroscopic signature of dimer formation but also additional contributions arising from matrix–solute interactions and should, consequently, be interpreted with appropriate caution when compared with gas phase measurements or theoretical predictions. In the following, we analyze how matrix effects modulate the observed dimer-formation frequency shifts and demonstrate that these perturbations do not compromise the identification of the structural binding motifs related to specific functional groups (OH, CH, OCO) of the system.

#### 3.2.1 OH related vibrations

For the *Δ*^*ν*OH^ shift obtained from gas phase reference (*Δ*^*ν*OH^_Gas_ = −487 cm^−1^) is slightly underestimated in argon (*Δ*^*ν*OH^_Ar_ = −478 cm^−1^) or overestimated in neon (*Δ*^*ν*OH^_Ne_ = −492 cm^−1^) MI-FTIR spectra. For obtaining the *Δ*^*δ*_ip_COH^ shift, we have to consider the resonance phenomenon (*cf.* Section 3.1) that occurs for the monomer both in argon (1305/1216 cm^−1^) and neon (1306/1218 cm^−1^) MI-FTIR spectra. Considering the dimer, we observe, with very low intensity, one band at 1371 cm^−1^ in argon and one band at 1373 cm^−1^ in neon matrices. If we adopt the higher-frequency component as the monomer reference (argon: 1306 cm^−1^; neon: 1305 cm^−1^), we obtain shifts in argon 
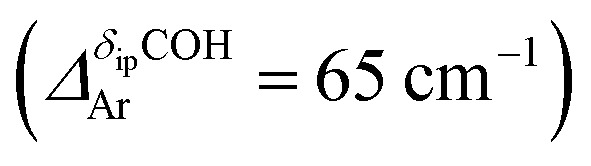
 and in neon 
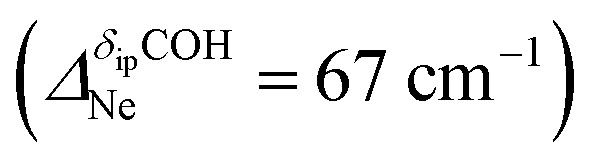
 MI-FTIR spectra that compare well with the corresponding gas phase reference 
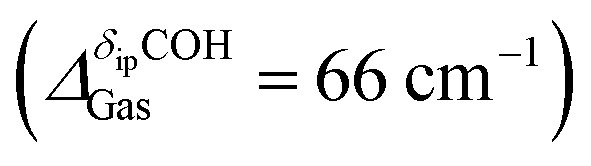
. The *Δ*^*δ*_oop_COH^ shift is slightly overestimated in both 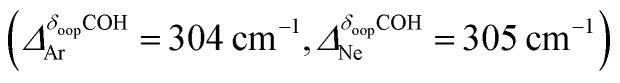 MI-FTIR spectra when compared to the gas phase reference 
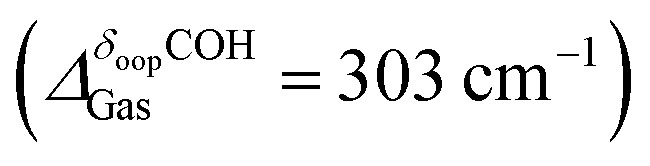
. In general, the discrepancies between the matrix and gas phase dimer-formation frequency shifts remain below 2% for all the OH-related vibrations.

#### 3.2.2 CH related vibrations

As discussed in Section 3.1, the *Δ*^*ν*CH^ shift is difficult to assess because of the strong overlap of monomer and dimer bands in the gas phase. Based on the commonly accepted assignment, the corresponding shift is very low (*Δ*^*ν*CH^_Gas_ = −3 cm^−1^). Due to the relatively low intensity of the bands corresponding to the *ν*CH vibration, we were not able to observe the dimer bands in our MI-FTIR spectra. We may refer to the work by Ito,^54^ who observed the dimer band at 2947 cm^−1^ in argon MI-FTIR spectra. This assignment would lead to a shift (*Δ*^*ν*CH^_Ar_ = −8 cm^−1^) that agrees qualitatively with the gas phase. Considering the *Δ*^*δ*_ip_CH^ shift, we cannot derive a shift for our MI-FTIR data, because we were not able to reliably observe the dimer band. Apparently, this was also the case in Ito's work, where no corresponding band is reported for the dimer. Gantenberg *et al.* report the *δ*_ip_CH of the dimer at 1445 cm^−1^, with a relative intensity of only 0.1% of the strongest band.^55^ Comparing the value reported by Gantenberg *et al.* to the monomer band in argon at 1381 cm^−1^ yields a shift of 
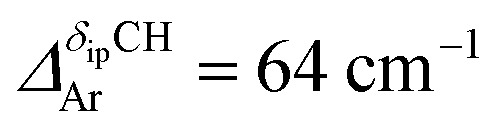
, which is considerably higher than the gas phase reference 
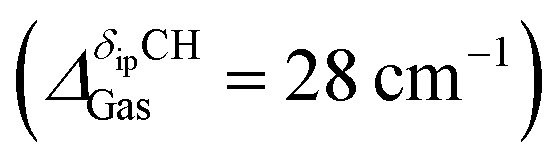
. It is very likely that this is not a matrix effect but a wrong assignment, suggesting that an observable dimer band may lie at a lower frequency than the one reported by Gantenberg *et al.* The *Δ*^*δ*_oop_CH^ shift, however, can be evaluated. Gantenberg *et al.* assigned the *δ*CH vibration to 1069.3 cm^−1^,^55^ and this assignment was later confirmed by Ito.^54^ In our spectra, we detect extremely weak bands that agree with this assignment. The shifts we obtain from our MI-FTIR experiments 
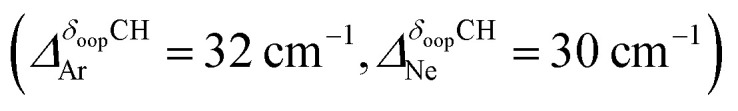
 are also in good agreement with gas phase reference 
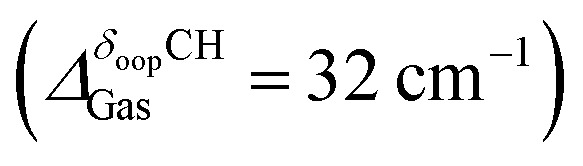
, suggesting a minor matrix effect. Nevertheless, it ultimately remains challenging to draw definitive conclusions regarding the influence of the matrix on the dimer-formation frequency shifts for the CH-related vibrations, primarily because their IR intensities are very low and the corresponding band assignments therefore remain unreliable.

#### 3.2.3 OCO related vibrations

The *Δ*^*ν*C=O^ shift as obtained from gas phase reference data (*Δ*^*ν*C=O^_Gas_ = −31 cm^−1^) is vividly overestimated in both (*Δ*^*ν*C=O^_Ar_ = −39 cm^−1^, *Δ*^*ν*C=O^_Ne_ = −36 cm^−1^) MI-FTIR spectra. For the *Δ*^*ν*C–O^ shift, the matrix effect is again negligible, where the shifts obtained from MI-FTIR spectra (*Δ*^*ν*C–O^_Ar_ = 123 cm^−1^, *Δ*^*ν*C–O^_Ne_ = 125 cm^−1^) are in excellent agreement with the gas phase reference (*Δ*^*ν*C–O^_Gas_ = 125 cm^−1^). Similarly, for the *Δ*^*δ*_ip_OCO^ shift, the shifts obtained from both 
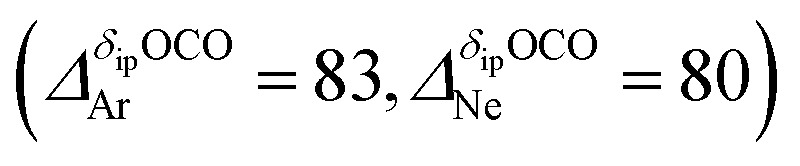
 MI-FTIR spectra closely match the gas phase reference 
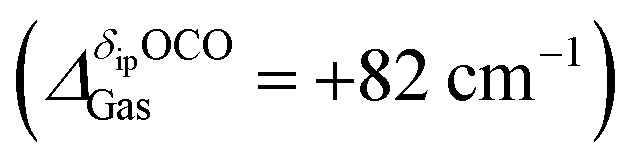
. With regard to the OCO-related vibrations, it should be noted that, in relative terms, the *Δ*^*ν*C=O^_Ar_ and *Δ*^*ν*C=O^_Ne_ shifts correspond to deviations of 28% and 17%, respectively. This identifies the spectral region exhibiting the most pronounced matrix effect on the dimer-formation frequency shifts. The *Δ*^*ν*C–O^ and *Δ*^*δ*_ip_OCO^ shifts from MI-FTIR spectra are again below 2% deviation from gas phase, consistent with the other regions.

### 3.3 Distinguishing conformers of the *trans*,*trans*-formic acid dimer *via* MI-FTIR spectroscopy

The various conformers that emerge from the clustering of formic acid have been studied extensively by computational approaches, including the dimer but also higher oligomers.^61–68^ In particular for the cyclic dimer, the double hydrogen-bond has been investigated extensively with respect to its influence on vibrational properties,^61^ and binding energies.^62^ Within MI-FTIR spectroscopy, primarily dimers have been observed,^54,69,70^ although trimers have also been reported.^71^ In the present work, we focus on the dimer conformers observable by MI-FTIR spectroscopy.

Our conformational sampling reveals several conformers that are worth considering, as illustrated in Fig. 3. Thermochemistry suggests that only the **A**^cyc^ conformer, *i.e.*, the doubly hydrogen-bonded “cyclic” dimer in *C*_2h_ symmetry, is thermodynamically favored. The **B** conformers, which correspond to singly hydrogen-bonded dimers in *C*_s_ symmetry, exhibit significantly higher Gibbs free energies of up to 4.4 kcal mol^−1^. We denote the **B**^open^ conformer as the “open” dimer because the two OH groups do not interact directly; instead, a single hydrogen bond forms between the OH group of one molecule and the carbonyl group of the other molecule. The **B**^bridge^ conformer is referred to as the “bridge” dimer because the OH group of one molecule forms a bridge between the OH group and the carbonyl group of the other molecule. Finally, the **B**^chain^ conformer is termed the “chain” dimer because the OH groups interact in a way that allows additional formic acid molecules to attach in a daisy-chain-like arrangement. One may also consider higher-energy conformers that do not exhibit a conventional hydrogen bond (the **C** and **D** conformers in Fig. 3). However, these structures lie far above the energy range thermally accessible at room temperature (∼0.6 kcal mol^−1^), so their populations are expected to be negligible.

Despite the unfavorable formation energy, MI-FTIR spectroscopy has been shown to distinguish some of these higher-energy conformers, as comprehensively demonstrated by Lopes *et al.*^70^ using N_2_ matrices (*cf.*Table 2). Based on their experiments and earlier argon matrix studies by Gantenberg,^55^ Ito,^54,71^ and Marushkevich *et al.*,^69,72^ Lopes *et al.*^70^ identified three hydrogen-bonded conformers of the *trans*–*trans* formic acid dimer conformers:^70^ the lowest-energy cyclic dimer with *C*_2h_ symmetry (**A**^cyc^ in Fig. 3), and two higher-energy dimers with *C*_s_ symmetry (**B**^open^ and **B**^chain^ in Fig. 3). Annealing at 32 K was found to increase the populations of the lowest (**A**^cyc^) and second-lowest (**B**^open^) energy conformers, while reducing the presence of higher-energy conformers.

**Table 2:** Absolute frequencies and dimer-formation frequency shifts (in cm^−1^) as observed in MI-FTIR spectra in nitrogen (*Δ*_N_2__), argon (*Δ*_Ar_), and neon (*Δ*_Ar_) compared to computational predictions from harmonic frequency calculations (*Δ*_DFT_) for different formic acid dimer conformers (**A**^cyc^, **B**^open^, **B**^bridge^, and **B**^chain^) in reference to the monomer (**M**)

Label		Matrix-isolation (literature)^a^				Matrix-isolation (this work)				Calculation (this work)^b^		
		N_2_	*Δ*_N_2__	Ar	*Δ*_Ar_	Ar	*Δ*_Ar_	Ne	*Δ*_Ne_	DFT	*Δ*_DFT_	Int.
**M**	*ν* **OH**	3528		3549		3550		3570		3862		104
**A** ^cyclic^				3072	−477	3073	−477	3078	−492	3299	−563	2573
**B** ^open^	“Free”	3509	−19	3540	−9	3537	−13	3564	−6	3854	−7	113
	“Bond”	3154	−374	3184	−365	3169	−381	3207	−363	3468	−394	1071
**B** ^bridge^	“Free”					3425	−125	3461	−109	3751	−111	504
	“Bond”							3310	−260	3602	−260	400
**B** ^chain^	“Free”	3518	−11	3544	−5	3537	−13	3564	−6	3855	−7	110
	“Bond”	3371	−158	3387	−162	3388	−162	3419	−151	3702	−160	602
**M**	*ν* **C–H**	2968		2955		2953		2939		3165		27
**A** ^cyclic^				2947*	−8					3171	6	303
**B** ^open^	“Free”	2951	−16							3150	−15	64
	“Bond”	2997	29							3206	41	11
**M**	*ν* **C=O**	1762		1768		1768		1774		1897		442
**A** ^cyclic^		1728	−34	1728	−40	1729	−40	1738	−36	1856	−42	996
**B** ^open^	“Free”	1745	−17	1748	−20	1746	−22	1755	−19	1872	−25	792
	“Bond”	1693	−69			1713	−55	1719	−55	1828	−69	150
**B** ^bridge^	“Free”					1775	7	1777	3	1902	5	364
	“Bond”					1734	−34	1736	−38	1860	−37	591
**B** ^chain^	“free”	1771	9	1774	6	1779	11	1779	5	1905	8	498
	“Bond”			1750	−18	1753	−15	1752	−22	1875	−23	398
**M**	*δ*_ip_ **CH**	1342		1383		1381		1380		1428		5
**A** ^cyclic^				1445*	62					1465	38	1
**M**	*δ*_ip_ **COH**	1265		1306		1306		1305		1336		29
**A** ^cyclic^		1375	110	1372	66	1371	65	1373	67	1437	102	79
**B** ^open^	“Free”	1293	28							1360	24	71
	“Bond”	1360	95							1415	80	26
**B** ^bridge^	“Free”									1330	−6	19
	“Bond”									1390	54	15
**B** ^chain^	“Free”									1301	−34	0
	“Bond”									1394	59	31
**M**	*ν* **C–O**	1119		1104		1104		1103		1184		278
**A** ^cyclic^		1227	108	1225	121	1227	123	1226	123	1314	130	368
**B** ^open^	“Free”	1150	31	1131	27	1131	27	1130	27	1208	24	288
	“Bond”	1181	62	1180	76	1180	76	1177	74	1269	86	236
**B** ^bridge^	“Free”							1114	11	1193	9	528
	“Bond”					1159	55	1171	68	1240	56	302
**B** ^chain^	“Free”									1151	−33	234
	“Bond”	1180	60	1154	50	1154	50			1239	55	322
**M**	*δ*_oop_ **CH**	1041		1038		1038		1036		1076		1
**A** ^cyclic^		1109	68	1069	31	1070	32	1066	30	1118	42	51
**B** ^open^	“Free”	1051	11							1092	16	4
	“Bond”									1115	39	1
**M**	*δ*_oop_ **COH**	672		635		636		638		681		169
**A** ^cyclic^		938	266	939	304	940	304	943	305	1018	338	208
**B** ^open^	“Free”	695	22	658	23	658	22	661	23	702	22	164
	“Bond”	877	205	858	223	868	232	891	253	933	252	130
**B** ^bridge^	“Free”					696	60	699	61	741	60	39
	“Bond”									865	185	239
**B** ^chain^	“Free”					611	−25	611	−27	654	−26	150
	“Bond”									808	127	155
**M**	*δ*_ip_ **OCO**	637		629		629		626		650		51
**A** ^cyclic^		722	85	712	83	712	83	707	81	740	90	51
**B** ^open^	“Free”	660	23							671	21	84
	“Bond”	680	43							704	55	36

a The reference MI-FTIR data obtained in N_2_ matrices were taken from Lopes *et al.*^70,73^ The reference MI-FTIR data in Ar matrices were compiled from the studies of Macoas *et al.*,^74^ Marushkevich *et al.*,^69,72^ Ito,^54,71^ and Gantenberg *et al.*^55^ For monomer bands split by matrix trapping sites, the corresponding frequencies were averaged when calculating dimerization-induced frequency shifts. Assignments marked with an asterisk (*) are uncertain and are discussed in the text. For direct comparison with previous studies, the conformers designated here as **A**^cyc^, **B**^open^, and **B**^chain^ correspond to the previously reported TT1, TT2, and TT5 structures, respectively.

b Harmonic calculations at *ω*B97X-3c level of theory. No scaling factors applied. Calculated absolute intensities (Int.) are given in km mol^−1^.

**Fig. 4:**
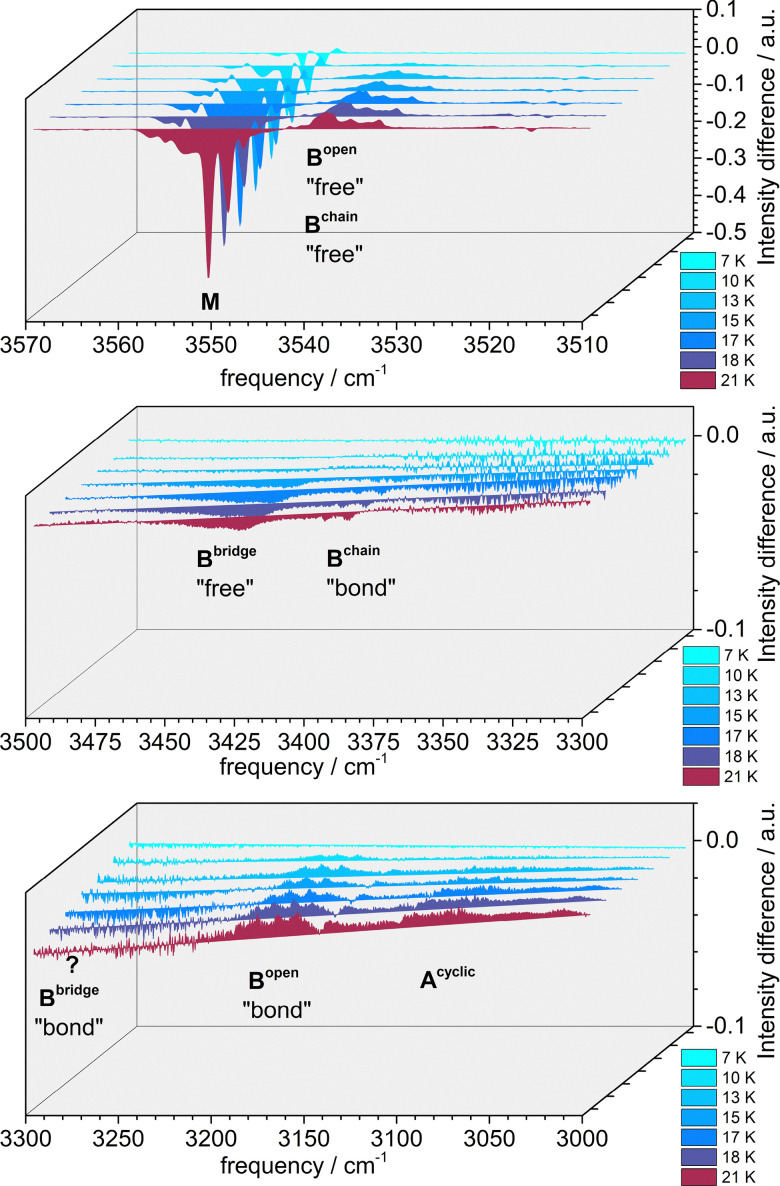
Matrix-isolation FTIR difference spectra obtained during the annealing procedure to visualize dimer formation in the *ν*OH region (3570–3000 cm^−1^). A 1 : 250 mixture of formic acid : argon was deposited at 5 K and subsequently annealed stepwise up to 24 K. Difference spectra were recorded with reference to the 5 K spectrum. M stands for monomer; the other labels correspond to different dimer conformers (see Fig. 3).

Fig. 4 shows the MI-FTIR spectrum in the region where the *ν*OH vibration is expected, for different annealing temperatures. Similar to Lopes *et al.*,^70^ we observe spectral changes upon annealing to 24 K in an argon matrix. As shown, we have assigned multiple bands to the formic acid dimer, corresponding to the various conformers listed in Fig. 3. This includes bands assigned to energetically less stable conformers of the dimer. The existence of such higher-energy conformers has previously been discussed by Madeja,^75^ who observed “polar” structures of the formic acid dimer in helium nanodroplets. Subsequent studies demonstrated that formic acid preferentially forms singly hydrogen-bonded dimers in this environment,^76^ while molecular dynamics simulations suggested that these structures are stabilized by kinetic trapping effects.^77^ A similar kinetic trapping mechanism may also account for the observation of higher-energy conformers in solid argon and neon matrices, as observed here.

Assigning the absolute band positions for conformers based on anharmonic calculations is not practical, due to the cost of such calculations. However, the various conformers can be identified based on the dimer-formation frequency shifts predicted by harmonic calculations using a cost-efficient DFT approach (*Δ*_DFT_), as discussed in Section 3.1 for the **A**^cyc^ conformer. Table 2 summarizes these dimer-formation frequency shifts as determined experimentally from MI-FTIR spectroscopy (*Δ*_Ar_, *Δ*_Ne_), and computationally from harmonic calculations (*Δ*_DFT_) for the **A** and **B** conformers of the dimer. In the following, we identify the *ν*OH, *ν*C=O, *ν*C–O, *δ*_ip_COH, and *δ*_oop_COH vibration as the most reliable and characteristic infrared-active modes to study cluster formation with respect to the carboxyl group. We limit the discussion to the argon MI-FTIR spectra, but the corresponding neon data are given in Table 2 for completeness. All relevant regions of the MI-FTIR spectra and their assignment are shown in the SI.

#### 3.3.1 OH-related vibrations

*ν*OH: the *Δ*^*ν*OH^ shifts span a wide range, with the largest shift being *Δ*^*ν*OH^_Ar_(**A**^cyc^) = −477 cm^−1^ for the “bond” *ν*OH vibration of the **A**^cyc^ conformer. This is in line with the strong hydrogen bond in the cyclic dimer. Note that the prediction of *Δ*^*ν*OH^_DFT_(**A**^cyc^) = −563 cm^−1^ is not good, most likely due to the strong anharmonicity in this particular case of the **A**^cyc^ conformer (*cf.* Section 3.1). The annealing experiments show a slight increase in the intensity of the corresponding band, indicating that the population of the **A**^cyc^ conformer increases (*cf.*Fig. 4). The dimer-formation frequency shifts of the “bond” *ν*OH vibrations of the **B** conformers successively become smaller from the **B**^open^ over the **B**^bridge^ to the **B**^chain^ conformer, indicating decreasing trend of the hydrogen bond strength. The trend starts with the *Δ*^*ν*OH^_Ar_(**B**^open^) = −381 cm^−1^ shift, which is in good agreement with the prediction of *Δ*^*ν*OH^_DFT_(**B**^open^) = −394 cm^−1^. This band shows increasing intensity in the annealing experiments, suggesting that the population of the **B**^open^ conformer increases. The predicted *Δ*^*ν*OH^_DFT_(**B**^bridge^) = −260 cm^−1^, which should be in the middle of the trend, could not be assigned in our argon spectra, also not after annealing. We were able to find the *Δ*^*ν*OH^_Ar_(**B**^chain^) = −162 cm^−1^ for the weakest hydrogen bond, in good agreement with the prediction of *Δ*^*ν*OH^_DFT_(**B**^chain^) = −160 cm^−1^. A small decrease is visible for the corresponding band in the annealing experiment, indicating that the population of the **B**^chain^ conformer increases.

The “free” *ν*OH vibrations of the **B** conformers behave differently than the above-mentioned ones. The “free” *ν*OH vibration of the **B**^open^ and the **B**^chain^ conformer cannot be distinguished and we find a common shift of *Δ*^*ν*OH^_Ar_(**B**^open^) = *Δ*^*ν*OH^_Ar_(**B**^chain^) = −13 cm^−1^ in qualitative agreement with the prediction of *Δ*^*ν*OH^_DFT_(**B**^open^) = *Δ*^*ν*OH^_DFT_(**B**^chain^) = −7 cm^−1^. In the **B**^open^ conformer, one OH group is “free” because it does not participate in a hydrogen bond. In the **B**^chain^ conformer, the OH groups are connected as a “chain”, and the OH group at the end of the chain behaves similarly to a “free” OH group. Consequently, both conformers exhibit *ν*OH vibrations similar to the *ν*OH of the monomer, aligning with the assumption of a weak or absent hydrogen-bond interaction. Here, the annealing experiments lead to a substantial increase in intensity. This behavior is most plausibly attributable to an increase in the population of the **B**^open^ conformer alone. In contrast, the population of the **B**^chain^ conformer was observed to decrease for the corresponding “bond” *Δ*^*ν*OH^_Ar_(**B**^chain^) shift, and this is most likely the case also here. The “free” *ν*OH vibration of the **B**^bridge^ conformer can be assigned to a band detected at 3425 cm^−1^ in argon, indicating a redshift of *Δ*^*ν*OH^_Ar_(**B**^bridge^) = −125 cm^−1^ in good agreement with the prediction of *Δ*^*ν*OH^_DFT_(**B**^bridge^) = −111 cm^−1^. These values suggest an intermediate hydrogen-bond strength, less robust than the **A**^cyc^ conformer, but stronger than in the **B**^open^ or **B**^chain^ conformer type. The **B**^bridge^ conformer has one OH group that takes part in two hydrogen bonds, *i.e.*, functioning as a “bridge” between the OH group and the C=O group in one formic acid unit. Thus, the vibration is somewhat between the “free” and “bond” vibrations. The annealing experiments show a slight decrease in intensity for this band, indicating a decrease in the population of the **B**^bridge^ conformer.

*δ*_ip_COH: we have discussed in Section 3.1 that the harmonic calculations overestimate the *Δ*^*δ*_ip_COH^(**A**^cyc^) shifts due to the intrinsic limitation of reproducing resonance phenomena. It can be expected that this also holds for all **B** conformers. From the predicted “bond” *δ*_ip_COH vibrations, we expect a decrease in the blueshift from the **B**^open^, over the **B**^bridge^ to the **B**^chain^ conformer. This is consistent with partial hydrogen bonding that becomes progressively weaker relative to the **A**^cyc^ conformer. For the “free” *δ*_ip_COH vibrations, the calculations predict a similar decreasing trend, reaching negative values, reflecting the even weaker hydrogen-bonding interactions. Although there are some bands in the argon and neon MI-FTIR spectra that may be assigned to these features, we refrain from doing so. Not only because we expect the harmonic calculation to be a poor prediction here, but also due to the very low intensities of these vibrations. Considering the annealing experiments, both the monomer (**M**) and the cyclic dimer (**A**^cyc^) slightly decrease upon annealing, while no other bands rise above the signal-to-noise threshold.

*δ*_oop_COH: similar to the “bond” *ν*OH vibrations, we observe large shifts for the “bond” *δ*_oop_COH vibrations, the largest being 
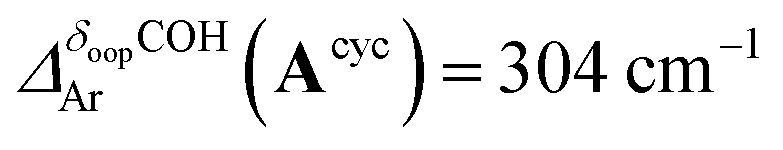
 followed by the 
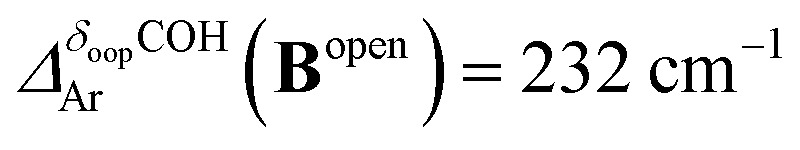
. The corresponding shifts for the **B**^bridge^ and **B**^chain^ conformers should decrease progressively in magnitude, according to the calculations. However, the bands were not detectable. In contrast, the “free” *δ*_oop_COH vibrations were all detectable yielding values of 
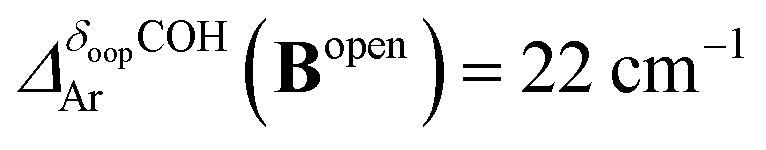
, 
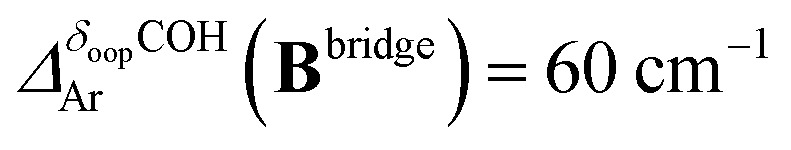
, and 
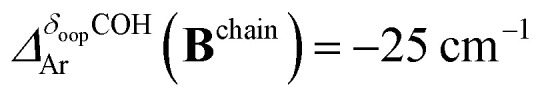
. Similar to the “free” *ν*OH vibrations, we observe that the **B**^open^ and **B**^chain^ are similar in magnitude, albeit with different directions. The larger shift predicted for the **B**^bridge^ conformer is consistent with the OH group functioning as a “bridge”, being somewhat in between the “free” and “bond” vibrations. Annealing experiments of these bands show minor changes, which are difficult to quantify due to their low intensities.

#### 3.3.2 CH-related vibrations

As discussed in Section 3.2, the expected *Δ*^*ν*CH^ shift for the **A**^cyc^ is very small, and the dimer band intensity is so weak that it is nearly unobservable, and we have to refer to literature data. While the *Δ*^*δ*_ip_CH^ shift is larger, it is practically not possible to obtain reliable data due to the low intensity. For the *Δ*^*δ*_oop_CH^ shift, we can obtain a value; however, this is merely evidence that the MI-FTIR spectra are capable of identifying very weak features, rather than a useful marker. Lopes *et al.* have reported 
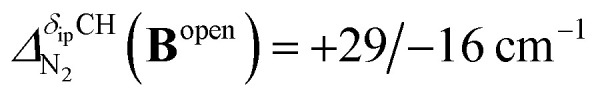
 and 
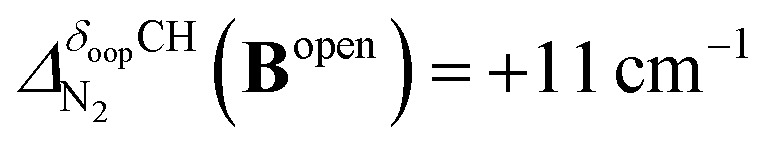
, which agrees with computational predictions 
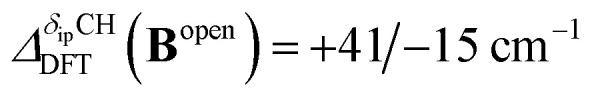
 and 
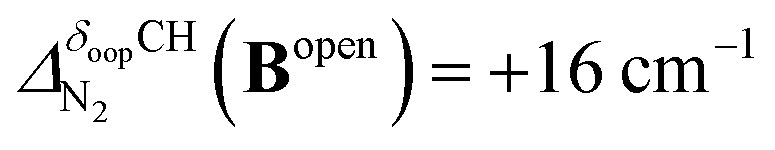
. However, the intensities of these bands are too low to assign them in our spectra. Ultimately, we regard all CH-related vibrations as generally unsuitable for probing other conformers as well. Annealing experiments do not change these conclusions; the bands remain mostly undetectable.

#### 3.3.3 OCO-related vibrations

**Fig. 5:**
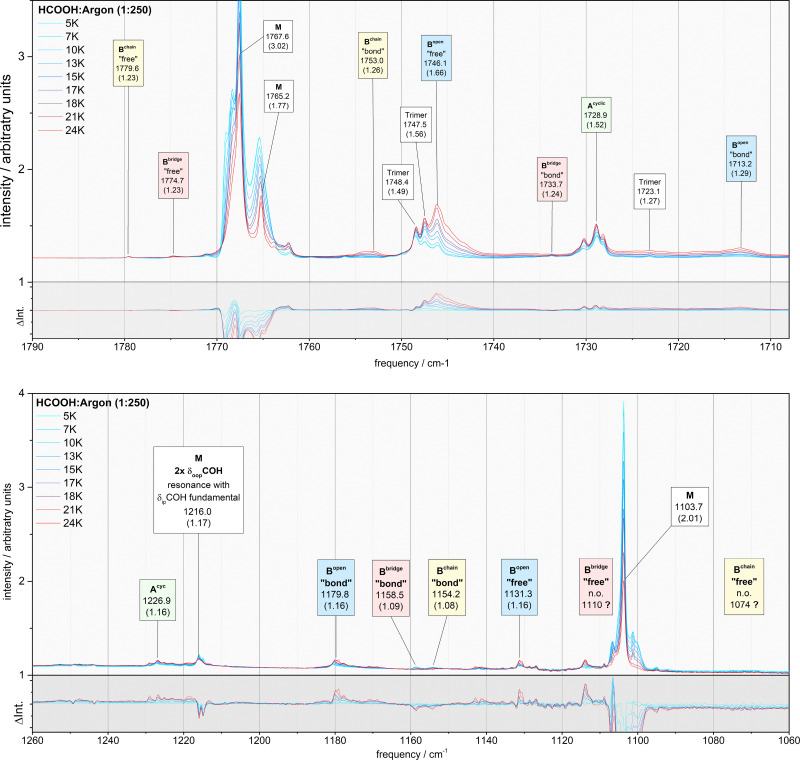
Matrix-isolation FTIR spectrum of formic acid (HCOOH) in argon. The panel displays the *ν*C=O (top) and the *ν*C–O (bottom) spectral regions recorded at different annealing temperatures. Spectral features attributable to the monomer (**M**, gray), the cyclic double-hydrogen-bonded dimer (**A**^cyc^, green), as well as several singly hydrogen-bonded dimer conformers (**B**^open^, blue; **B**^bridge^, orange; **B**^chain^, yellow) can be identified. For the latter conformers, both “free” and hydrogen-“bonded” variants of the *ν*C=O vibrational mode are observed. The trimer assignments are tentative.

*ν*C=O: as shown in Fig. 5, the *ν*C=O vibrations exhibit a narrower range of dimer-formation frequency shifts than the *ν*OH vibrations, reflecting a lesser sensitivity of the carbonyl stretching frequency to hydrogen-bond formation. Nevertheless, the observed trends mirror the hierarchy observed above for the *ν*OH vibration. Considering the “bond” *ν*C=O vibrations, the largest shift is *Δ*^*ν*C=O^_Ar_(**B**^open^) = −55 cm^−1^, followed by *Δ*^*ν*C=O^_Ar_(**A**^cyc^) = −40 cm^−1^, *Δ*^*ν*C=O^_Ar_(**B**^bridge^) = −34 cm^−1^, and *Δ*^*ν*C=O^_Ar_(**B**^chain^) = −15 cm^−1^. Interestingly, the **A**^cyc^ conformer lies between the **B** conformers, *i.e.*, it does not show the largest shift of all, in contrast to all other vibrations. In the annealing experiments, the bands related to the **A**^cyc^ and **B**^open^ conformers increase significantly, while we also detect a slight increase of the **B**^chain^ “bond” vibration (which was not detected for *ν*OH). Considering the “free” *ν*C=O vibrations, the only redshift is *Δ*^*ν*C=O^_Ar_(**B**^open^) = −22 cm^−1^, while the other two are small blueshifts of *Δ*^*ν*C=O^_Ar_(**B**^bridge^) = 7 cm^−1^ and *Δ*^*ν*C=O^_Ar_(**B**^chain^) = 11 cm^−1^. These bands are of low intensity and very close to the broader, more intense monomer band. Here, the annealing experiments help to distinguish the dimer bands from the monomer bands. Again, we observe that the bands related to the **B**^open^, **A**^cyc^ increase in intensity during annealing, but for **B**^chain^ no changes are visible, more in line with the results from *ν*OH vibration. From a practical perspective, it may be highlighted that changes in the population of dimer conformers during annealing are most conveniently observed in the *ν*C=O stretching region, as all conformers are observed in a confined spectral range, simplifying normalization between the spectra recorded at different annealing steps.

*ν*C–O: Fig. 5 shows the region of the *ν*C–O vibration. For the “bond” *ν*C–O vibration we observe a pronounced shift of *Δ*^*ν*C–O^_Ar_(**A**^cyc^) = 122 cm^−1^. The second largest shift of this type is *Δ*^*ν*C–O^_Ar_(**B**^open^) = 76 cm^−1^. The remaining **B** conformers are expected to have almost the same shift from the harmonic calculation. Marushkevich *et al.*^69^ report from their argon matrix-isolation experiments, a shift of *Δ*^*ν*C–O^_Ar,_(**B**^bridge^) = 56 cm^−1^ and of *Δ*^*ν*C–O^_Ar,_(**B**^chain^) = 51 cm^−1^. We observe similar features with a shift of *Δ*^*ν*C–O^_Ar_(**B**^bridge^) = 55 cm^−1^ and of *Δ*^*ν*C–O^_Ar_(**B**^chain^) = 50 cm^−1^. The corresponding bands vanish with annealing, suggesting that both the **B**^bridge^ and the **B**^chain^ are lost during annealing. This is consistent with observation in other spectral regions.

For the “free” *ν*C–O vibration, the situation is a bit different. Here, the shifts are of much smaller magnitude, and we can only identify the shift of *Δ*^*ν*C–O^_Ar_(**B**^open^) = 27 cm^−1^. If we consider again the observations by Marushkevich *et al.*,^69^ they report a shift of *Δ*^*ν*C–O^_Ar,Ref_(**B**^bridge^) = 7 cm^−1^ and of *Δ*^*ν*C–O^_Ar,Ref_(**B**^chain^) = −27 cm^−1^. These observations are plausible when compared to our corresponding calculated shifts of *Δ*^*ν*C–O^_DFT_(**B**^bridge^) = 9 cm^−1^ and *Δ*^*ν*C–O^_DFT_(**B**^chain^) = −27 cm^−1^. However, in our experiments, the bands are barely visible, neither before nor after annealing. Generally, the annealing experiments show an increase in both bands related to the *ν*C–O vibration of **B**^open^.

*δ*_ip_OCO: for the sake of completeness, we may mention here that besides the 
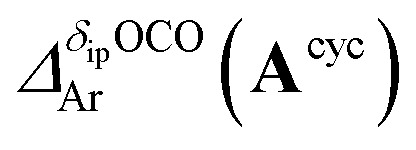
 shift, we were not able to evaluate shifts of the other conformers. Here, Lopes *et al.* have reported 
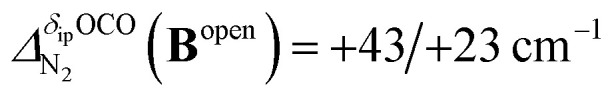
, which agrees with computational predictions 
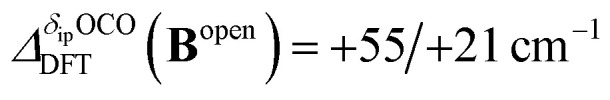
. However, due to the low intensity of bands related to this vibration, they are not detectable in our experiments.

### 3.4 The formic acid trimer

In the present study, we did not calculate trimer conformers. However, we observe several bands that were previously assigned to the formic acid trimer by Ito using matrix-isolation.^54,71^ This trimer assignment was revised later by jet-expansion experiments.^56,78^ We may mention the *ν*C=O region, where Ito assigned three features at 1746, 1747, and 1748 cm^−1^ to the trimer. In the present work, the band at 1746 cm^−1^ is assigned to the **B**^open^ dimer (*cf.*Fig. 5). Addition of a further formic acid molecule to this structure would yield one of the trimer motifs proposed by Ito for these bands. Similar motifs have been also proposed by Roy and Thakkar.^65^ It is therefore likely that this pattern arises from a mixture of dimer and trimer species with similar structural motifs. Further computational studies of the trimer conformational space would be required to validate this interpretation. In addition, the proximity of the bands is comparable to the spectral resolution used in this study, so further experiments may be necessary to resolve these features more clearly. Such investigations are beyond the scope of the present work. Nevertheless, the ease with which these supposedly trimer-related bands appear highlights the capability of matrix-isolation to probe cluster formation.

## 4 Conclusion

MI-FTIR experiments can identify structural motifs among different conformers of the formic acid dimer. These motifs manifest in the magnitude and direction of dimer-formation frequency shifts, which follow clear trends. We show that harmonic frequency calculations based on DFT (*e.g.*, using the *ω*B97X-3c functional) are sufficient to predict the direction and approximate magnitude of the frequency shifts associated with dimerization. Higher level of theory in harmonic frequency calculations at CCSD(T)-F12/cc-PVTZ-F12 provides slight improvements on the prediction of the dimer frequency shifts compared to DFT, but are computationally too demanding for larger organic systems important for NPF. Anharmonic calculations of vibrational frequencies improve absolute frequency predictions but do not significantly improve predictions of dimer-formation frequency shifts. However, subtleties such as resonances, as in the case of the *δ*_ip_COH vibration, cannot be resolved solely through harmonic frequency calculations, underscoring the importance of the anharmonic analysis presented in our previous work. In general, the calculated values follow the same qualitative trends as the experimental data. However, the experimentally observed apparent dimer-formation shifts include contributions from matrix-induced shifts of the underlying monomer and dimer vibrations. Although this effect is small enough not to interfere with the analysis of cluster formation, dedicated theoretical studies to compute matrix frequency shifts remain desirable to quantify their contributions more rigorously.

Using the harmonic predictions of the dimer-formation frequency shifts, we investigated the different vibrational modes for their ability to identify the structural motifs in cluster formation. In the *ν*OH and *ν*C=O regions, redshifts are relatively large for doubly hydrogen-bonded groups, moderate for singly hydrogen-bonded groups, and small for free OH and C=O groups. The *ν*C=O region is conveniently located within a spectral window of about 100 cm^−1^, making it well suited for an initial assessment of cluster formation. This is particularly advantageous in annealing experiments, where slight baseline variations occur between spectra. In such cases, baseline correction is required, for example by normalizing to a chosen anchor wavenumber. This approach works reliably when applied over a relatively narrow spectral range. In contrast, the *ν*OH region of the monomer and dimer spans over 500 cm^−1^ and exhibits comparatively low intensities for the dimer bands, making it a secondary choice for studying organic cluster formation. However, in both regions, the harmonic approximations of the dimer-formation frequency shifts and the matrix-induced frequency shifts exhibit considerable deviations from gas-phase reference values, which could be problematic when larger systems are studied.

The *ν*C–O and *δ*_oop_COH regions, in contrast, provide a surprisingly rich source of information on different dimer structures, as the theoretical predictions are highly accurate and the matrix-induced frequency shifts are almost negligible. In contrast to the stretching regions discussed above, the dimer bands are blueshifted here. The *ν*C–O region extends over roughly 100 cm^−1^ and allows for an equally reliable initial assessment as the *ν*C=O region. The *δ*_oop_COH region spans approximately 300 cm^−1^ and can serve as a secondary diagnostic region, similar to the *ν*OH region. However, both regions have slightly lower band intensities compared to *ν*C=O, making the identification of all vibrational bands of all conformers challenging.

All vibrational modes involving the CH group (*ν*CH, *δ*_ip_CH, and *δ*_oop_CH) exhibit very low intensities, such that even assignment of the monomer bands becomes challenging. These modes are therefore not recommended for studies of cluster formation. While monomer and cyclic dimer bands can be observed in the *δ*_ip_COH and *δ*_ip_OCO regions, their low intensities make straightforward assignment of other dimer conformers difficult. Although these regions are not particularly useful for identifying cluster formation, they may still serve to confirm the presence of the most stable conformer.

Thermochemistry suggests that conformers with more hydrogen-bonded groups should be more populated than those with fewer such interactions. This expectation is only partially consistent with our matrix annealing experiments, which should drive the kinetically trapped system closer to its equilibrium state. While the population of the **A**^**cyclic**^ conformer (double hydrogen bond) increases slightly as expected, the population of the **B**^open^ conformer (single hydrogen bond) also increases. This indicates the formation of less-stable dimeric motifs in the matrix. The populations of the **B**^bridge^ and **B**^chain^ conformers either remain constant or decrease. During annealing, the monomer concentration decreases due to evaporation into the vacuum and dimer formation, while the corresponding dimer bands increase. Combined with the observation that the population of the lowest-energy conformer does not grow substantially, whereas higher-energy conformers increase markedly, we conclude that matrix-isolation slows the kinetics of dimerization and enables direct visualization of the earliest steps of new particle formation by trapping non-equilibrium conformers, which could also be important in atmospheric clustering processes.

Atmospheric cluster formation and growth are typically modeled by calculating the free energy gain associated with assembling a cluster from isolated monomers. A major challenge is linking such models to experimental observations. MI-FTIR provides a qualitative lower bound on the accessible conformer space because formation rates in the atmospheric gas-phase at ambient temperature are expected to exceed those achievable in a cryogenic matrix. Continued development of computational approaches for predicting matrix-isolated conformer populations will further strengthen this connection. However, the simple normal-mode decomposition for manual assignment of labels from the chemist notation becomes increasingly tedious as the conformational space expands beyond our simple formic acid model system with a manageable number of conformers, highlighting the need for further automation.

Overall, the structural insights from MI-FTIR spectroscopy provide molecular-level information on the earliest steps of organic new particle formation that cannot be obtained by conventional mass spectrometry. For formic acid, the cyclic dimer with two hydrogen bonds is confirmed as the most stable species and therefore the most relevant for thermodynamic modeling of new particle formation. It can be expected that similar motifs are also essential for larger organic acids. We identified *ν*C=O, *ν*OH, *ν*C–O, and *δ*_oop_COH as the major vibrational modes to study cluster organic formation. The present study, therefore, establishes a foundational reference for future investigations of mixtures of formic acid with sulfuric acid and other atmospheric organic molecules.

## Author contributions

Dennis F. Dinu: conceptualization, data curation, formal analysis, investigation, methodology, validation, visualization, writing – original draft, writing – review & editing. Vincent Enders: investigation, validation, writing – review & editing. Julius Stolze: investigation. Lukas Meinschad: data curation, formal analysis, investigation, methodology, validation, visualization, writing – review & editing. Jonas Schlagin: formal analysis, investigation, validation, visualization, writing – review & editing. Klaus R. Liedl: funding acquisition, resources, writing – review and editing. Guntram Rauhut: validation, software, methodology, writing – review & editing. Thomas Loerting: funding acquisition, resources. Hinrich Grothe: conceptualization, funding acquisition, resources. Maren Podewitz: writing – review and editing. Dominik Stolzenburg: conceptualization, investigation, funding acquisition, project administration, supervision, writing – original draft, writing – review & editing.

## Conflicts of interest

There are no conflicts to declare.

## Supplementary Material

CP-028-D6CP01819J-s001

## Data Availability

The data supporting this article have been included as part of the supplementary information (SI). Supplementary information is available. See DOI: https://doi.org/10.1039/d6cp01819j.

## References

[cit1] Kulmala M., Kontkanen J., Junninen H., Lehtipalo K., Manninen H. E., Nieminen T., Petäjä T., Sipilä M., Schobesberger S., Rantala P., Franchin A., Jokinen T., Järvinen E., Äijälä M., Kangasluoma J., Hakala J., Aalto P. P., Paasonen P., Mikkilä J., Vanhanen J., Aalto J., Hakola H., Makkonen U., Ruuskanen T., Mauldin R. L., Duplissy J., Vehkamäki H., Bäck J., Kortelainen A., Riipinen I., Kurtén T., Johnston M. V., Smith J. N., Ehn M., Mentel T. F., Lehtinen K. E. J., Laaksonen A., Kerminen V.-M., Worsnop D. R. (2013). Science.

[cit2] Kerminen V.-M., Chen X., Vakkari V., Petäjä T., Kulmala M., Bianchi F. (2018). Environ. Res. Lett..

[cit3] Stolzenburg D., Cai R., Blichner S. M., Kontkanen J., Zhou P., Makkonen R., Kerminen V.-M., Kulmala M., Kangasluoma J. (2023). Rev. Mod. Phys..

[cit4] Kulmala M., Dada L., Daellenbach K. R., Yan C., Stolzenburg D., Kontkanen J., Ezhova E., Hakala S., Tuovinen S., Kokkonen T. V., Kurppa M., Cai R., Zhou Y., Yin R., Baalbaki R., Chan T., Chu B., Deng C., Fu Y., Ge M., He H., Heikkinen L., Junninen H., Liu Y., Lu Y., Nie W., Rusanen A., Vakkari V., Wang Y., Yang G., Yao L., Zheng J., Kujansuu J., Kangasluoma J., Petäjä T., Paasonen P., Järvi L., Worsnop D., Ding A., Liu Y., Wang L., Jiang J., Bianchi F., Kerminen V.-M. (2021). Faraday Discuss..

[cit5] Gordon H., Kirkby J., Baltensperger U., Bianchi F., Breitenlechner M., Curtius J., Dias A., Dommen J., Donahue N. M., Dunne E. M., Duplissy J., Ehrhart S., Flagan R. C., Frege C., Fuchs C., Hansel A., Hoyle C. R., Kulmala M., Kürten A., Lehtipalo K., Makhmutov V., Molteni U., Rissanen M. P., Stozkhov Y., Tröstl J., Tsagkogeorgas G., Wagner R., Williamson C., Wimmer D., Winkler P. M., Yan C., Carslaw K. S. (2017). J. Geophys. Res.: Atmos..

[cit6] Kirkby J., Curtius J., Almeida J., Dunne E., Duplissy J., Ehrhart S., Franchin A., Gagné S., Ickes L., Kürten A., Kupc A., Metzger A., Riccobono F., Rondo L., Schobesberger S., Tsagkogeorgas G., Wimmer D., Amorim A., Bianchi F., Breitenlechner M., David A., Dommen J., Downard A., Ehn M., Flagan R. C., Haider S., Hansel A., Hauser D., Jud W., Junninen H., Kreissl F., Kvashin A., Laaksonen A., Lehtipalo K., Lima J., Lovejoy E. R., Makhmutov V., Mathot S., Mikkilä J., Minginette P., Mogo S., Nieminen T., Onnela A., Pereira P., Petäjä T., Schnitzhofer R., Seinfeld J. H., Sipilä M., Stozhkov Y., Stratmann F., Tomé A., Vanhanen J., Viisanen Y., Vrtala A., Wagner P. E., Walther H., Weingartner E., Wex H., Winkler P. M., Carslaw K. S., Worsnop D. R., Baltensperger U., Kulmala M. (2011). Nature.

[cit7] Almeida J., Schobesberger S., Kürten A., Ortega I. K., Kupiainen-Määttä O., Praplan A. P., Adamov A., Amorim A., Bianchi F., Breitenlechner M., David A., Dommen J., Donahue N. M., Downard A., Dunne E., Duplissy J., Ehrhart S., Flagan R. C., Franchin A., Guida R., Hakala J., Hansel A., Heinritzi M., Henschel H., Jokinen T., Junninen H., Kajos M., Kangasluoma J., Keskinen H., Kupc A., Kurtén T., Kvashin A. N., Laaksonen A., Lehtipalo K., Leiminger M., Leppä J., Loukonen V., Makhmutov V., Mathot S., McGrath M. J., Nieminen T., Olenius T., Onnela A., Petäjä T., Riccobono F., Riipinen I., Rissanen M., Rondo L., Ruuskanen T., Santos F. D., Sarnela N., Schallhart S., Schnitzhofer R., Seinfeld J. H., Simon M., Sipilä M., Stozhkov Y., Stratmann F., Tomé A., Tröstl J., Tsagkogeorgas G., Vaattovaara P., Viisanen Y., Virtanen A., Vrtala A., Wagner P. E., Weingartner E., Wex H., Williamson C., Wimmer D., Ye P., Yli-Juuti T., Carslaw K. S., Kulmala M., Curtius J., Baltensperger U., Worsnop D. R., Vehkamäki H., Kirkby J. (2013). Nature.

[cit8] Kirkby J., Duplissy J., Sengupta K., Frege C., Gordon H., Williamson C., Heinritzi M., Simon M., Yan C., Almeida J., Tröstl J., Nieminen T., Ortega I. K., Wagner R., Adamov A., Amorim A., Bernhammer A.-K., Bianchi F., Breitenlechner M., Brilke S., Chen X., Craven J., Dias A., Ehrhart S., Flagan R. C., Franchin A., Fuchs C., Guida R., Hakala J., Hoyle C. R., Jokinen T., Junninen H., Kangasluoma J., Kim J., Krapf M., Kürten A., Laaksonen A., Lehtipalo K., Makhmutov V., Mathot S., Molteni U., Onnela A., Peräkylä O., Piel F., Petäjä T., Praplan A. P., Pringle K., Rap A., Richards N. A. D., Riipinen I., Rissanen M. P., Rondo L., Sarnela N., Schobesberger S., Scott C. E., Seinfeld J. H., Sipilä M., Steiner G., Stozhkov Y., Stratmann F., Tomé A., Virtanen A., Vogel A. L., Wagner A. C., Wagner P. E., Weingartner E., Wimmer D., Winkler P. M., Ye P., Zhang X., Hansel A., Dommen J., Donahue N. M., Worsnop D. R., Baltensperger U., Kulmala M., Carslaw K. S., Curtius J. (2016). Nature.

[cit9] Tröstl J., Chuang W. K., Gordon H., Heinritzi M., Yan C., Molteni U., Ahlm L., Frege C., Bianchi F., Wagner R., Simon M., Lehtipalo K., Williamson C., Craven J. S., Duplissy J., Adamov A., Almeida J., Bernhammer A.-K., Breitenlechner M., Brilke S., Dias A., Ehrhart S., Flagan R. C., Franchin A., Fuchs C., Guida R., Gysel M., Hansel A., Hoyle C. R., Jokinen T., Junninen H., Kangasluoma J., Keskinen H., Kim J., Krapf M., Kürten A., Laaksonen A., Lawler M., Leiminger M., Mathot S., Möhler O., Nieminen T., Onnela A., Petäjä T., Piel F. M., Miettinen P., Rissanen M. P., Rondo L., Sarnela N., Schobesberger S., Sengupta K., Sipilä M., Smith J. N., Steiner G., Tomè A., Virtanen A., Wagner A. C., Weingartner E., Wimmer D., Winkler P. M., Ye P., Carslaw K. S., Curtius J., Dommen J., Kirkby J., Kulmala M., Riipinen I., Worsnop D. R., Donahue N. M., Baltensperger U. (2016). Nature.

[cit10] Wang M., Kong W., Marten R., He X.-C., Chen D., Pfeifer J., Heitto A., Kontkanen J., Dada L., Kürten A., Yli-Juuti T., Manninen H. E., Amanatidis S., Amorim A., Baalbaki R., Baccarini A., Bell D. M., Bertozzi B., Bräkling S., Brilke S., Murillo L. C., Chiu R., Chu B., Menezes L.-P. D., Duplissy J., Finkenzeller H., Gonzalez-Carracedo L., Granzin M., Guida R., Hansel A., Hofbauer V., Krechmer J., Lehtipalo K., Lamkaddam H., Lampimäki M., Lee C. P., Makhmutov V., Marie G., Mathot S., Mauldin R. L., Mentler B., Müller T., Onnela A., Partoll E., Petäjä T., Philippov M., Pospisilova V., Ranjithkumar A., Rissanen M., Rörup B., Scholz W., Shen J., Simon M., Sipilä M., Steiner G., Stolzenburg D., Tham Y. J., Tomé A., Wagner A. C., Wang D. S., Wang Y., Weber S. K., Winkler P. M., Wlasits P. J., Wu Y., Xiao M., Ye Q., Zauner-Wieczorek M., Zhou X., Volkamer R., Riipinen I., Dommen J., Curtius J., Baltensperger U., Kulmala M., Worsnop D. R., Kirkby J., Seinfeld J. H., El-Haddad I., Flagan R. C., Donahue N. M. (2020). Nature.

[cit11] He X.-C., Tham Y. J., Dada L., Wang M., Finkenzeller H., Stolzenburg D., Iyer S., Simon M., Kürten A., Shen J., Rörup B., Rissanen M., Schobesberger S., Baalbaki R., Wang D. S., Koenig T. K., Jokinen T., Sarnela N., Beck L., Almeida J., Amanatidis S., Amorim A., Ataei F., Baccarini A., Bertozzi B., Bianchi F., Brilke S., Caudillo L., Chen D., Chiu R., Chu B., Dias A., Ding A., Dommen J., Duplissy J., El-Haddad I., Carracedo L. G., Granzin M., Hansel A., Heinritzi M., Hofbauer V., Junninen H., Kangasluoma J., Kemppainen D., Kim C., Kong W., Krechmer J. E., Kvashnin A., Laitinen T., Lamkaddam H., Lee C. P., Lehtipalo K., Leiminger M., Li Z., Makhmutov V., Manninen H. E., Marie G., Marten R., Mathot S., Mauldin R. L., Mentler B., Möhler O., Müller T., Nie W., Onnela A., Petäjä T., Pfeifer J., Philippov M., Ranjithkumar A., Saiz-Lopez A., Salma I., Scholz W., Schuchmann S., Schulze B., Steiner G., Stozkhov Y., Tauber C., Tomé A., Thakur R. C., Väisänen O., Vazquez-Pufleau M., Wagner A. C., Wang Y., Weber S. K., Winkler P. M., Wu Y., Xiao M., Yan C., Ye Q., Ylisirniö A., Zauner-Wieczorek M., Zha Q., Zhou P., Flagan R. C., Curtius J., Baltensperger U., Kulmala M., Kerminen V.-M., Kurten T., Donahue N. M., Volkamer R., Kirkby J., Worsnop D. R., Sipilä M. (2021). Science.

[cit12] Shen J., Russell D. M., DeVivo J., Kunkler F., Baalbaki R., Mentler B., Scholz W., Yu W., Caudillo-Plath L., Sommer E., Ahongshangbam E., Alfaouri D., Almeida J., Amorim A., Beck L. J., Beckmann H., Berntheusel M., Bhattacharyya N., Canagaratna M. R., Chassaing A., Cruz-Simbron R., Dada L., Duplissy J., Gordon H., Granzin M., Schute L. G., Heinritzi M., Iyer S., Klebach H., Krüger T., Kürten A., Lampimäki M., Liu L., Lopez B., Martinez M., Morawiec A., Onnela A., Peltola M., Rato P., Reza M., Richter S., Rörup B., Sebastian M. K., Simon M., Surdu M., Tamme K., Thakur R. C., Tomé A., Tong Y., Top J., Umo N. S., Unfer G., Vettikkat L., Weissbacher J., Xenofontos C., Yang B., Zauner-Wieczorek M., Zhang J., Zheng Z., Baltensperger U., Christoudias T., Flagan R. C., Haddad I. E., Junninen H., Möhler O., Riipinen I., Rohner U., Schobesberger S., Volkamer R., Winkler P. M., Hansel A., Lehtipalo K., Donahue N. M., Lelieveld J., Harder H., Kulmala M., Worsnop D. R., Kirkby J., Curtius J., He X.-C. (2024). Nature.

[cit13] Zhao B., Donahue N. M., Zhang K., Mao L., Shrivastava M., Ma P.-L., Shen J., Wang S., Sun J., Gordon H., Tang S., Fast J., Wang M., Gao Y., Yan C., Singh B., Li Z., Huang L., Lou S., Lin G., Wang H., Jiang J., Ding A., Nie W., Qi X., Chi X., Wang L. (2024). Nature.

[cit14] Junninen H., Ehn M., Petäjä T., Luosujärvi L., Kotiaho T., Kostiainen R., Rohner U., Gonin M., Fuhrer K., Kulmala M., Worsnop D. R. (2010). Atmos. Meas. Tech..

[cit15] Jokinen T., Sipilä M., Junninen H., Ehn M., Lönn G., Hakala J., Petäjä T., Mauldin III R. L., Kulmala M., Worsnop D. R. (2012). Atmos. Chem. Phys..

[cit16] Riva M., Rantala P., Krechmer J. E., Peräkylä O., Zhang Y., Heikkinen L., Garmash O., Yan C., Kulmala M., Worsnop D., Ehn M. (2019). Atmos. Meas. Tech..

[cit17] Riva M., Ehn M., Li D., Tomaz S., Bourgain F., Perrier S., George C. (2019). Anal. Chem..

[cit18] Cai R., Mikkilä J., Bengs A., Koirala M., Mikkilä J., Holm S., Juuti P., Meder M., Partovi F., Shcherbinin A., Worsnop D., Ehn M., Kangasluoma J. (2024). Anal. Chem..

[cit19] Hoffmann T., Bandur R., Marggraf U., Linscheid M. (1998). J. Geophys. Res.: Atmos..

[cit20] Zhou S., Rivera-Rios J. C., Keutsch F. N., Abbatt J. P. D. (2018). Atmos. Meas. Tech..

[cit21] Russell L. M. (2003). Environ. Sci. Technol..

[cit22] Russell L. M., Bahadur R., Hawkins L. N., Allan J., Baumgardner D., Quinn P. K., Bates T. S. (2009). Atmos. Environ..

[cit23] Takahama S., Johnson A., Russell L. M. (2013). Aerosol Sci. Technol..

[cit24] Surdu M., Calmer R., Timarac-Popović J., Penn T., Luhmann N., Hiesberger J., Vukićević V., Alvino Démolis E., Favre L., Dönmez B., Bešić H., Kanellopulos K., Schmid S., Lafleur J. P., Takahama S., Schmale J. (2026). Sci. Adv..

[cit25] Bork N., Du L., Reiman H., Kurtén T., Kjaergaard H. G. (2014). J. Phys. Chem. A.

[cit26] Gadermann M., Vollmar D., Signorell R. (2007). Phys. Chem. Chem. Phys..

[cit27] Khare P., Kumar N., Kumari K. M., Srivastava S. S. (1999). Rev. Geophys..

[cit28] Millet D. B., Baasandorj M., Farmer D. K., Thornton J. A., Baumann K., Brophy P., Chaliyakunnel S., De Gouw J. A., Graus M., Hu L., Koss A., Lee B. H., Lopez-Hilfiker F. D., Neuman J. A., Paulot F., Peischl J., Pollack I. B., Ryerson T. B., Warneke C., Williams B. J., Xu J. (2015). Atmos. Chem. Phys..

[cit29] Goldstein A. H., Galbally I. E. (2007). Environ. Sci. Technol..

[cit30] Elm J., Myllys N., Kurtén T. (2017). J. Phys. Chem. A.

[cit31] Bianchi F., Kurtén T., Riva M., Mohr C., Rissanen M. P., Roldin P., Berndt T., Crounse J. D., Wennberg P. O., Mentel T. F., Wildt J., Junninen H., Jokinen T., Kulmala M., Worsnop D. R., Thornton J. A., Donahue N., Kjaergaard H. G., Ehn M. (2019). Chem. Rev..

[cit32] Kavouras I. G., Mihalopoulos N., Stephanou E. G. (1998). Nature.

[cit33] Dada L., Baalbaki R., Yao L., Xia M., Hakala S., Tham Y. J., Weidinger T., Kerminen V.-M., Yan C., Jokinen T., Salma I., Kulmala M. (2025). Aerosol Sci. Technol..

[cit34] Fang X., Hu M., Shang D., Tang R., Shi L., Olenius T., Wang Y., Wang H., Zhang Z., Chen S., Yu X., Zhu W., Lou S., Ma Y., Li X., Zeng L., Wu Z., Zheng J., Guo S. (2020). Environ. Sci. Technol. Lett..

[cit35] Zhang R., Li Y., Zhao J., Aridjis-Olivos B., Zhao L., Kowalewski V., Kabir M., Johnson N. M., Nielsen E. R., Brooks S. D., Zhang Y., Vedlitz A., Porter W., North S. W., Li W., Young M. W., Seinfeld J. H., Wang Y., Wang Y. (2026). Science.

[cit36] Elm J. (2019). ACS Omega.

[cit37] Elm J., Kubečka J., Besel V., Jääskeläinen M. J., Halonen R., Kurtén T., Vehkamäki H. (2020). J. Aerosol Sci..

[cit38] Kubečka J., Besel V., Neefjes I., Knattrup Y., Kurtén T., Vehkamäki H., Elm J. (2023). ACS Omega.

[cit39] Halonen R. (2022). J. Aerosol Sci..

[cit40] Badger R. M., Bauer S. H. (1937). J. Chem. Phys..

[cit41] Rozenberg M., Loewenschuss A. (2009). J. Phys. Chem. A.

[cit42] Rozenberg M., Loewenschuss A., Nielsen C. J. (2014). J. Phys. Chem. A.

[cit43] Chang Y. T., Yamaguchi Y., Miller W. H., Schaefer H. F. (1987). J. Am. Chem. Soc..

[cit44] Heger M., Suhm M. A., Mata R. A. (2014). J. Chem. Phys..

[cit45] Heger M. , PhD thesis, Georg-August-University, Göttingen, 2016

[cit46] Fischer T. L., Bödecker M., Schweer S. M., Dupont J., Lepère V., Zehnacker-Rentien A., Suhm M. A., Schröder B., Henkes T., Andrada D. M., Balabin R. M., Singh H. K., Bhattacharyya H. P., Sarma M., Käser S., Töpfer K., Vazquez-Salazar L. I., Boittier E. D., Meuwly M., Mandelli G., Lanzi C., Conte R., Ceotto M., Dietrich F., Cisternas V., Gnanasekaran R., Hippler M., Jarraya M., Hochlaf M., Viswanathan N., Nevolianis T., Rath G., Kopp W. A., Leonhard K., Mata R. A. (2023). Phys. Chem. Chem. Phys..

[cit47] Dinu D. F., Meinschad L., Schlagin J., Enders V., Podewitz M., Stolzenburg D., Rauhut G., Loerting T., Grothe H., Liedl K. R. (2026). Phys. Chem. Chem. Phys..

[cit48] Pracht P., Grimme S., Bannwarth C., Bohle F., Ehlert S., Feldmann G., Gorges J., Müller M., Neudecker T., Plett C., Spicher S., Steinbach P., Wesołowski P. A., Zeller F. (2024). J. Chem. Phys..

[cit49] Bannwarth C., Ehlert S., Grimme S. (2019). J. Chem. Theory Comput..

[cit50] Müller M., Hansen A., Grimme S. (2023). J. Chem. Phys..

[cit51] Neese F. (2025). Wiley Interdiscip. Rev.: Comput. Mol. Sci..

[cit52] Oenen K., Dinu D. F., Liedl K. R. (2024). J. Chem. Phys..

[cit53] Meinschad L., Oenen K., Dinu D. F., Liedl K. R. (2025). J. Mol. Spectrosc..

[cit54] Ito F. (2008). J. Chem. Phys..

[cit55] Gantenberg M., Halupka M., Sander W. (2000). Chem. – Eur. J..

[cit56] Nejad A. , PhD thesis, Georg-August-University, Göttingen, 2022

[cit57] Adler T. B., Knizia G., Werner H.-J. (2007). J. Chem. Phys..

[cit58] Peterson K. A., Adler T. B., Werner H.-J. (2008). J. Chem. Phys..

[cit59] Freytes M., Hurtmans D., Kassi S., Liévin J., Vander Auwera J., Campargue A., Herman M. (2002). Chem. Phys..

[cit60] Georges R., Freytes M., Hurtmans D., Kleiner I., Vander Auwera J., Herman M. (2004). Chem. Phys..

[cit61] Kalescky R., Kraka E., Cremer D. (2013). Mol. Phys..

[cit62] Kalescky R., Kraka E., Cremer D. (2014). J. Chem. Phys..

[cit63] Turi L. (1996). J. Phys. Chem..

[cit64] Stein M., Sauer J. (1997). Chem. Phys. Lett..

[cit65] Roy A. K., Thakkar A. J. (2004). Chem. Phys. Lett..

[cit66] Roy A. K., Thakkar A. J. (2004). Chem. Phys. Lett..

[cit67] Roy A. K., Thakkar A. J. (2005). Chem. Phys..

[cit68] Ziemczonek L., Wróblewski T., Karwasz G. P. (2006). Opt. Appl..

[cit69] Marushkevich K., Siltanen M., Räsänen M., Halonen L., Khriachtchev L. (2011). J. Phys. Chem. Lett..

[cit70] Lopes S., Fausto R., Khriachtchev L. (2018). J. Chem. Phys..

[cit71] Ito F. (2015). J. Mol. Struct..

[cit72] Marushkevich K., Khriachtchev L., Lundell J., Domanskaya A., Räsänen M. (2010). J. Phys. Chem. A.

[cit73] Lopes S., Domanskaya A. V., Fausto R., Räsänen M., Khriachtchev L. (2010). J. Chem. Phys..

[cit74] Maçôas E. M., Lundell J., Pettersson M., Khriachtchev L., Fausto R., Räsänen M. (2003). J. Mol. Spectrosc..

[cit75] Madeja F., Havenith M., Nauta K., Miller R. E., Chocholoušová J., Hobza P. (2004). J. Chem. Phys..

[cit76] Meyer K. A. E., Davies J. A., Ellis A. M. (2020). Phys. Chem. Chem. Phys..

[cit77] Ellis A. M., Davies J. A., Yurtsever E., Calvo F. (2022). J. Chem. Phys..

[cit78] Meyer K. A. E., Suhm M. A. (2017). J. Chem. Phys..

