# Supplementary material for: Vibrational spectra of formic acid and its dimer: II. Towards identifying structural motifs in atmospheric organic clustering
Source: Phys Chem Chem Phys. 2026 Jul 22. Online ahead of print. doi: 10.1039/d6cp01819j (PMC13398933; doi:10.1039/d6cp01819j)
Supplement: CP-OLF-D6CP01819J-s001 [file CP-OLF-D6CP01819J-s001.pdf]

Supplementary Information (SI) for Physical Chemistry Chemical Physics.
This journal is © the Owner Societies 2026

Vibrational Spectra of Formic Acid and Its Dimer: II. Towards Identifying Structural Motifs in Atmospheric Organic Clustering - Supplementary Information

Dennis F. Dinu, Vincent Enders, Julius Stolze,
Lukas Meinschad, Jonas Schlagin, Klaus R. Liedl, Guntram Rauhut,
Thomas Loerting, Hinrich Grothe, Maren Podewitz, Dominik Stolzenburg

July 8, 2026

Contents

1 Assignment of the Annealed Matrix Isolation FTIR Spectra from this Work 3
1.1 ν**OH** region [3600–3500 cm^{-1}] . . . 3
1.2 ν**OH** region [3500–3300 cm^{-1}] . . . 4
1.3 ν**OH** region [3300–3000 cm^{-1}] . . . 5
1.4 ν**CH** region [3000–2900 cm^{-1}] . . . 6
1.5 ν**C=O** region [1800–1700 cm^{-1}] . . . 7
1.6 δ_{ip}**CH** & δ_{ip}**COH** region [1450–1280 cm^{-1}] . . . 8
1.7 ν**C–O** region [1300–1050 cm^{-1}] . . . 9
1.8 δ_{oop}**CH** region [1100–1000 cm^{-1}] . . . 10
1.9 δ_{oop}**COH** region [1000–800 cm^{-1}] . . . 11
1.10 δ_{oop}**COH** & δ_{ip}**OCO** region [800–600 cm^{-1}] . . . 12
1.11 Difference spectra in argon . . . 13

2 Computational Thermochemistry of Dimer-formation 14

3 Dimer-formation Frequency Shifts (A-cyclic) using Different Computational Methods 15

4 Dimer-formation Frequency Shifts for the A-cylcic, B-open, B-Bridge, B-chain Conformers 16

5 Normal Mode Decomposition of Formic Acid Dimer Conformers 17
5.1 B-Open conformer . . . 19
5.2 B-Bridge conformer . . . 21
5.3 B-Chain conformer . . . 23

6 Supplementary References 25

1 Assignment of the Annealed Matrix Isolation FTIR Spectra from this Work

1.1 νOH region [3600–3500 cm^{-1}]

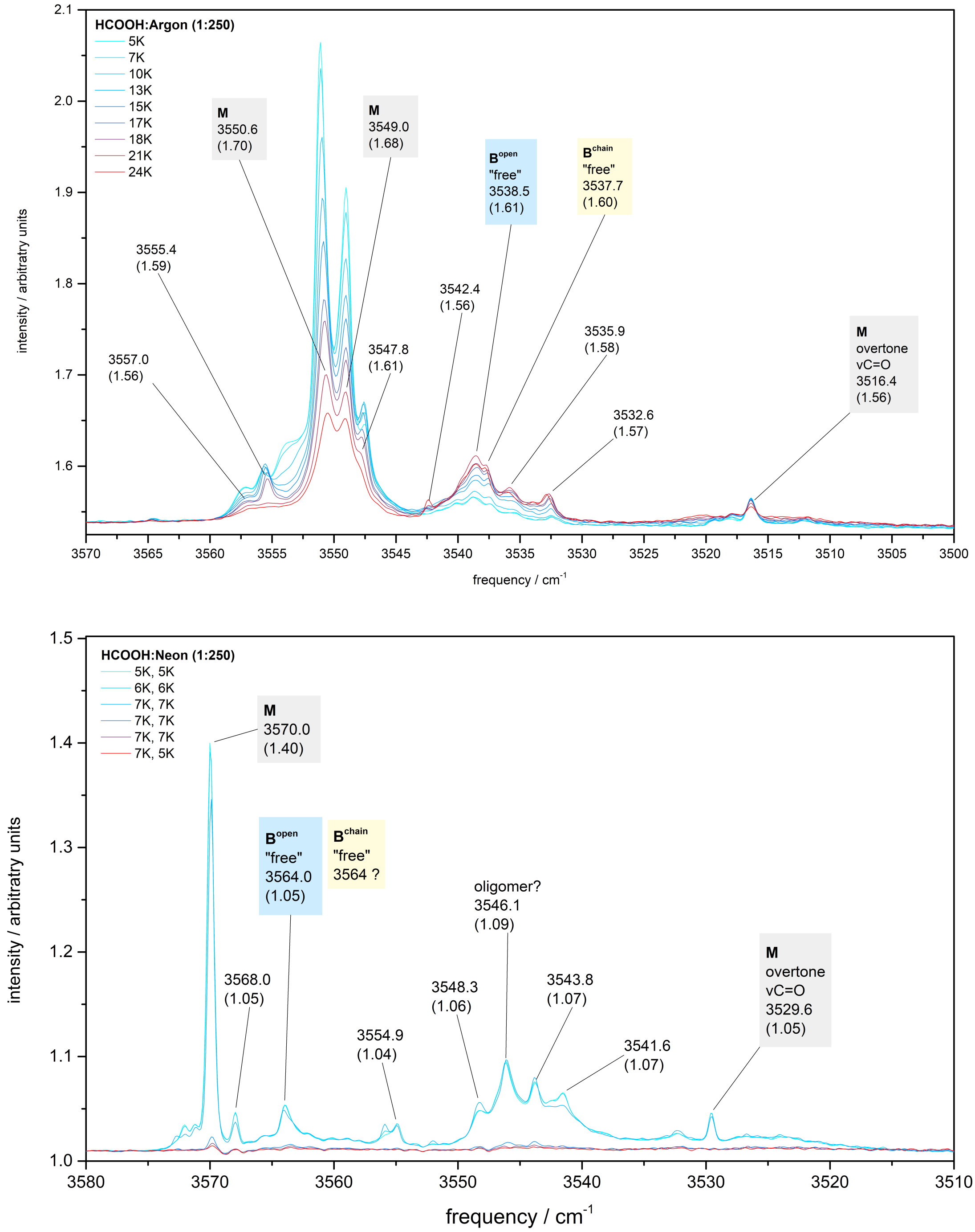

In argon, spectra were always recorded at the annealing temperature; therefore, only a single temperature is reported (e.g. 7 K). In neon, however, the sample was recooled after the final annealing step to 5 K before spectral acquisition, and both the annealing and measurement temperatures are therefore given (e.g. 7 K, 5 K).

1.2 νOH region [3500–3300 cm^{-1}]

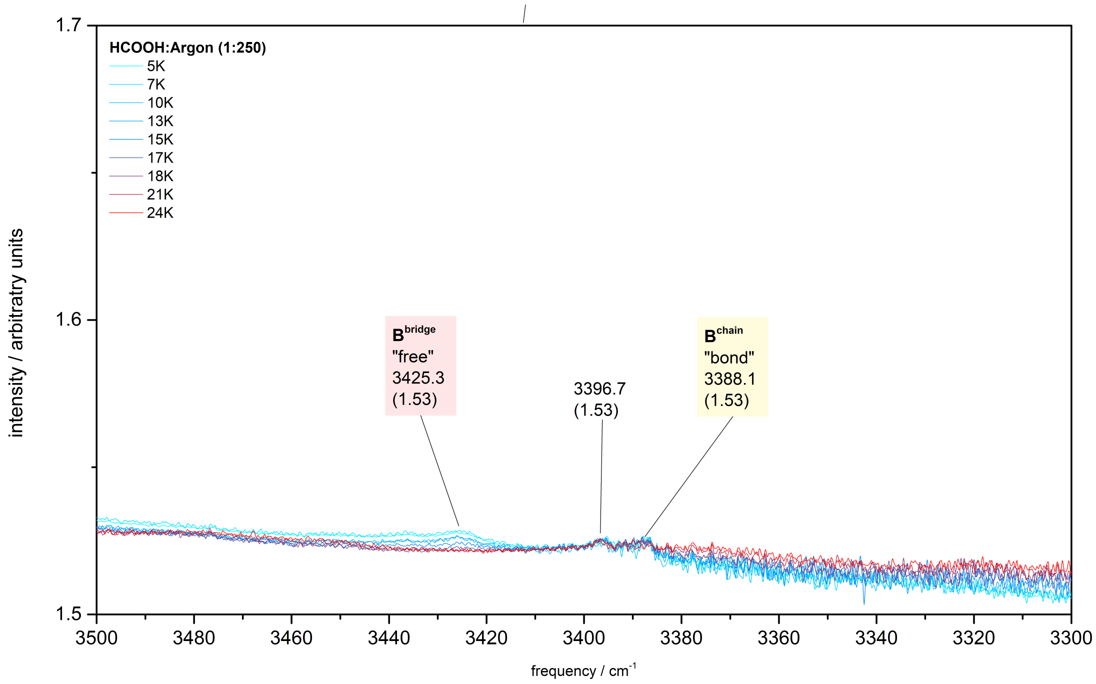

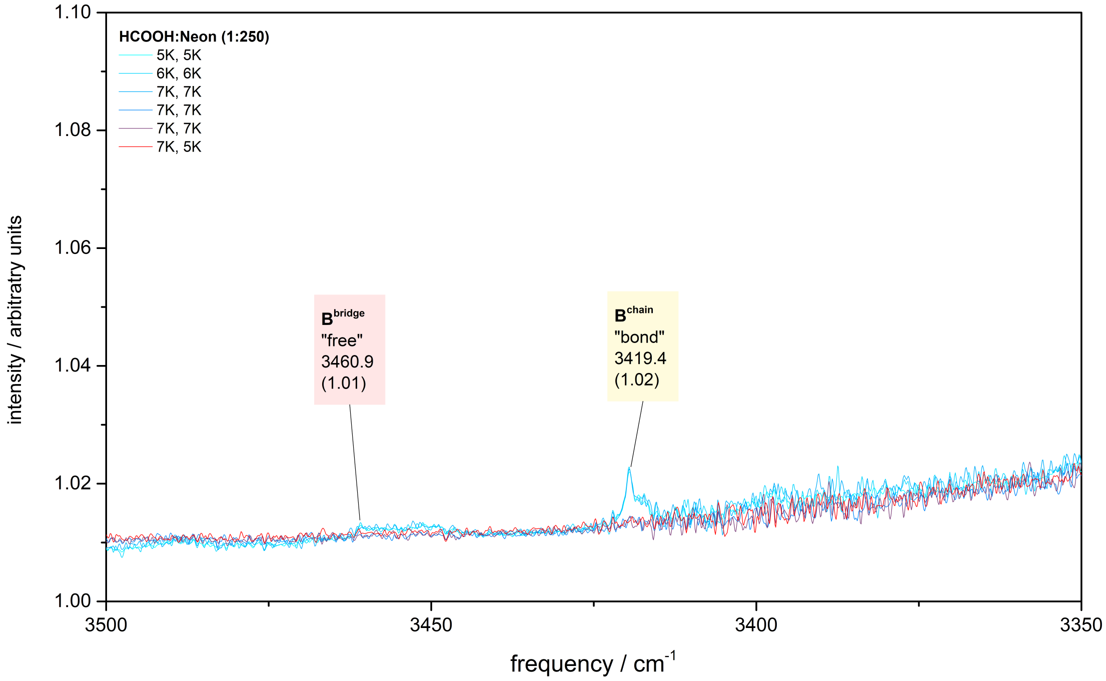

1.3 νOH region [3300–3000 cm^{-1}]

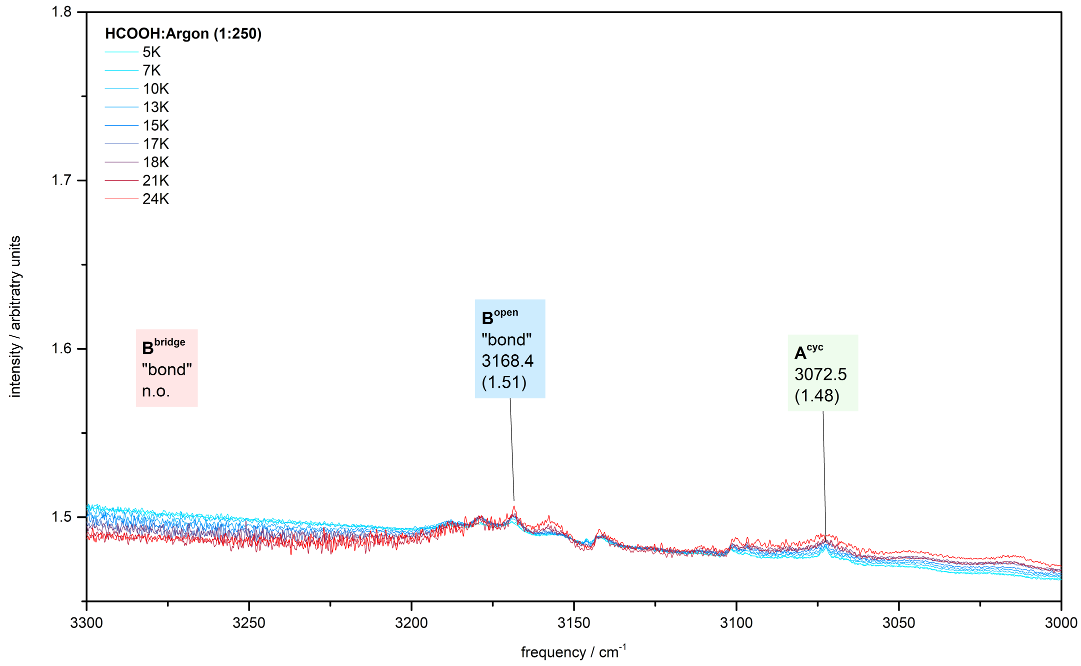

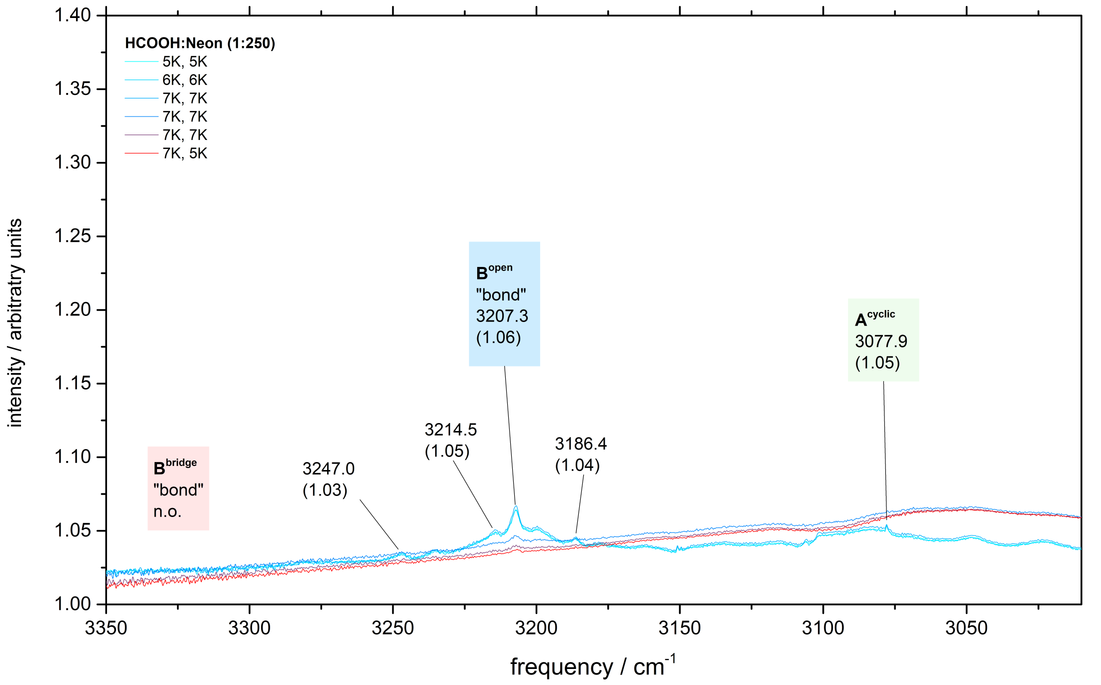

1.4 νCH region [3000–2900 cm^{-1}]

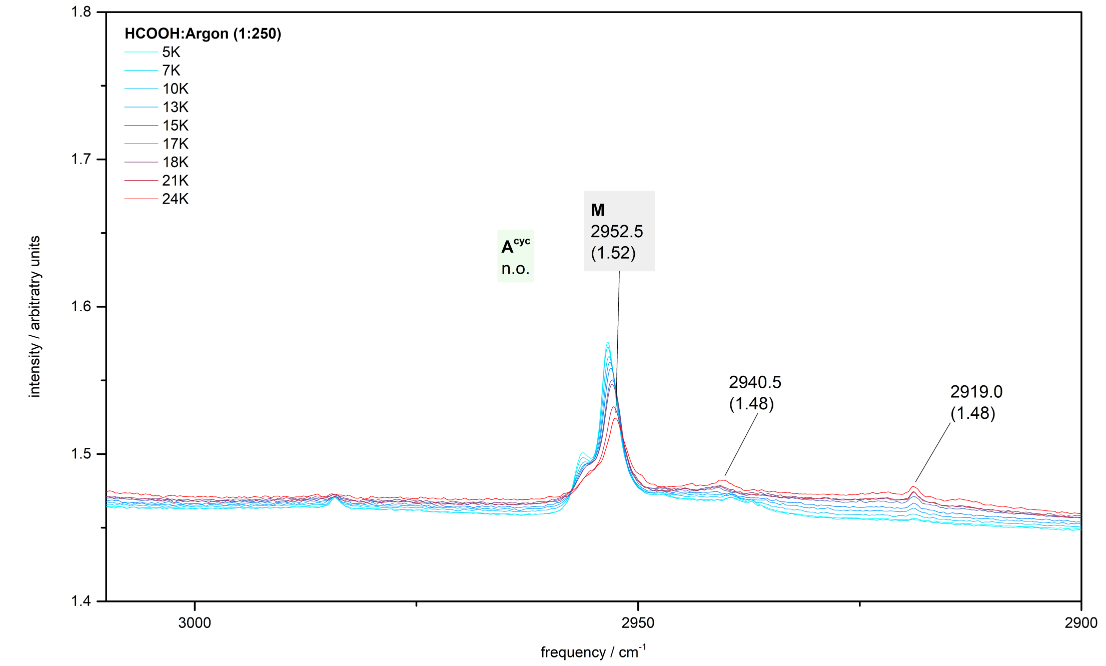

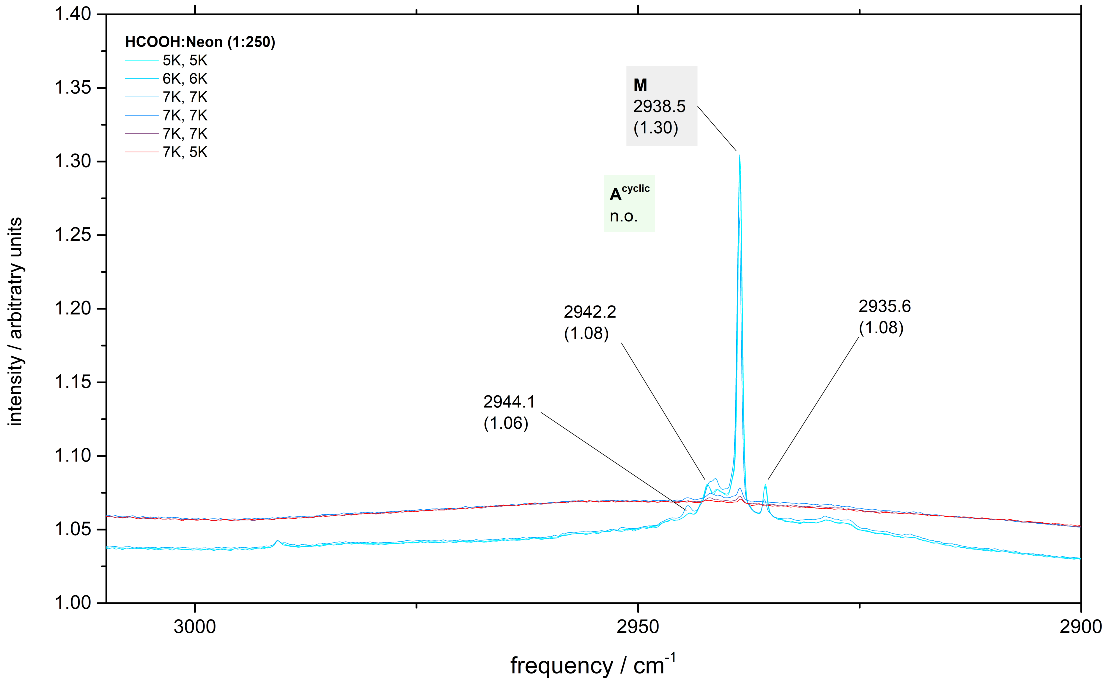

1.5 νC=O region [1800–1700 cm^{-1}]

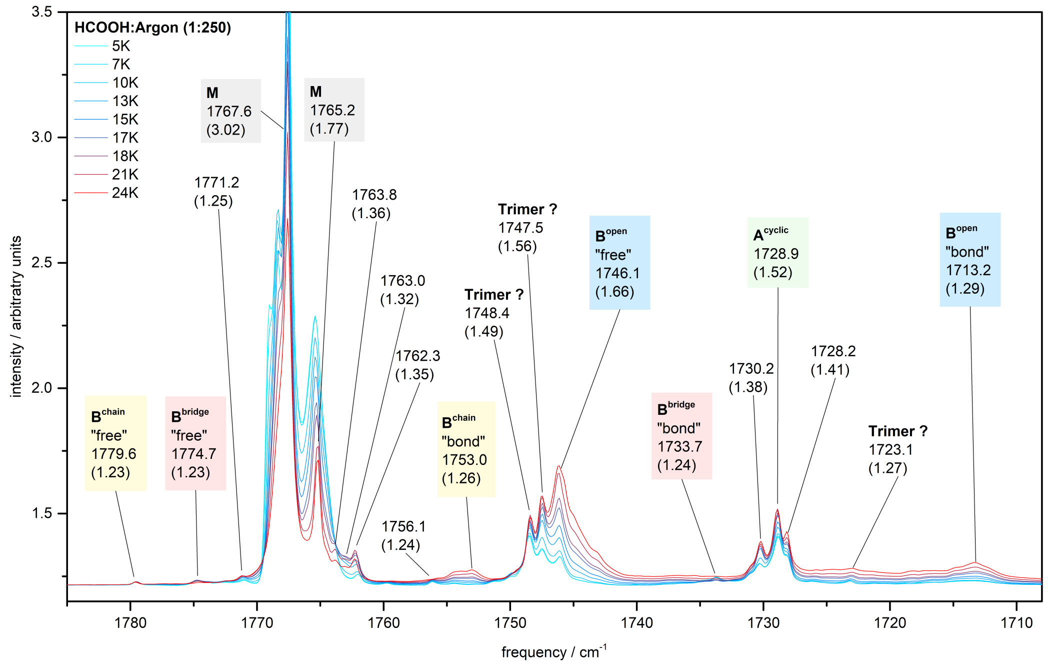

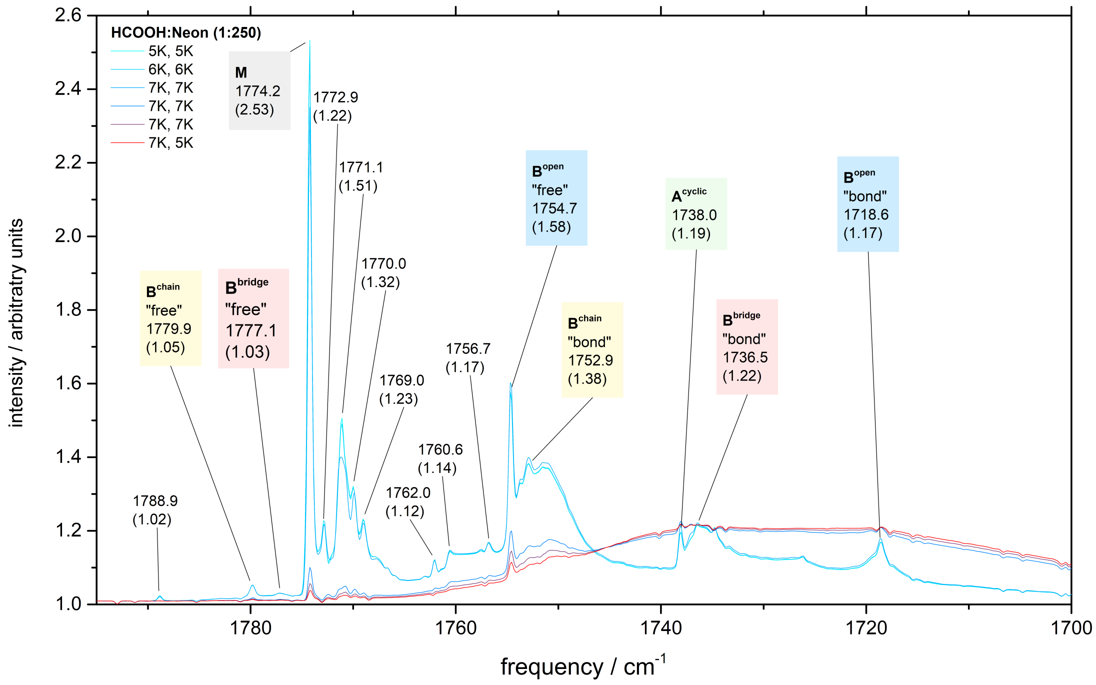

1.6 δ_{ip}CH & δ_{ip}COH region [1450–1280 cm^{-1}]

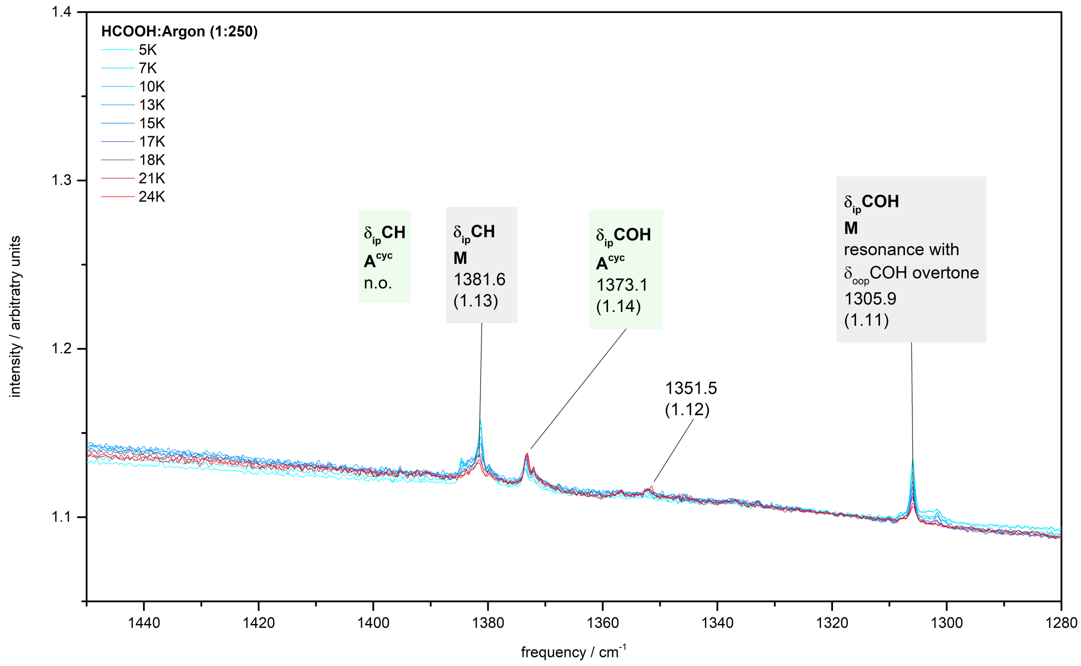

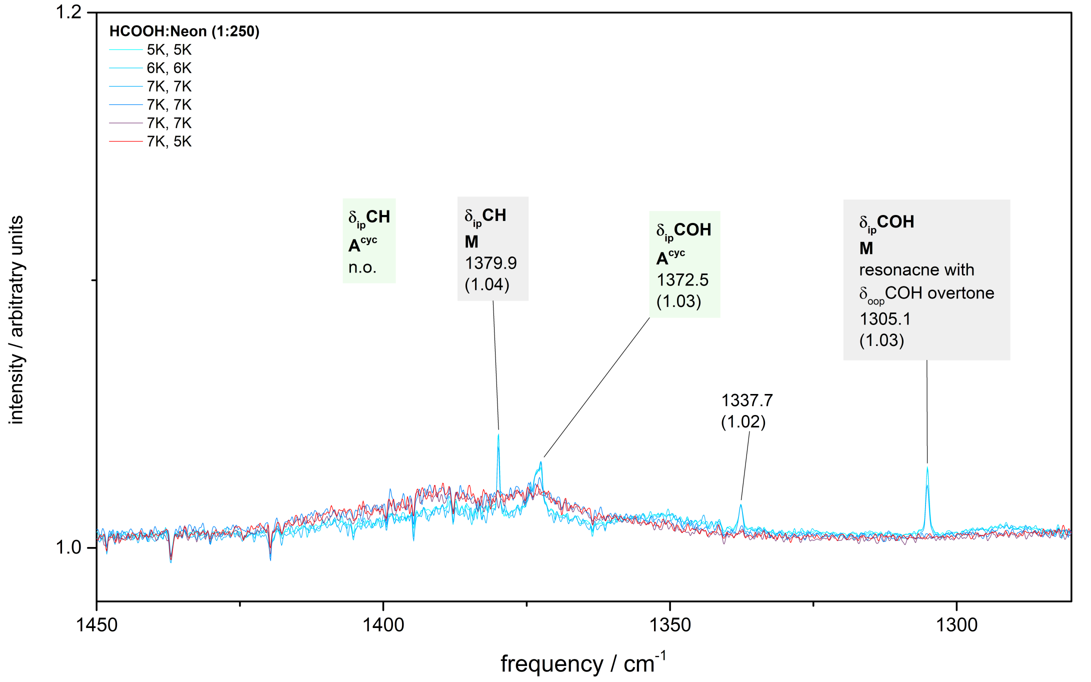

1.7 νC–O region [1300–1050 cm^{-1}]

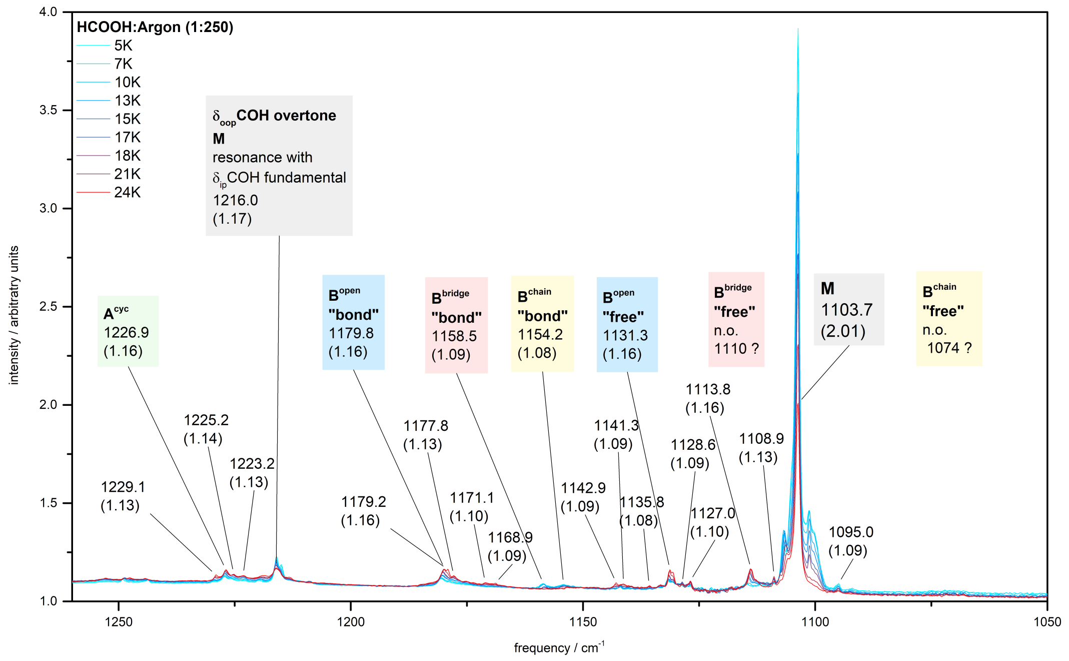

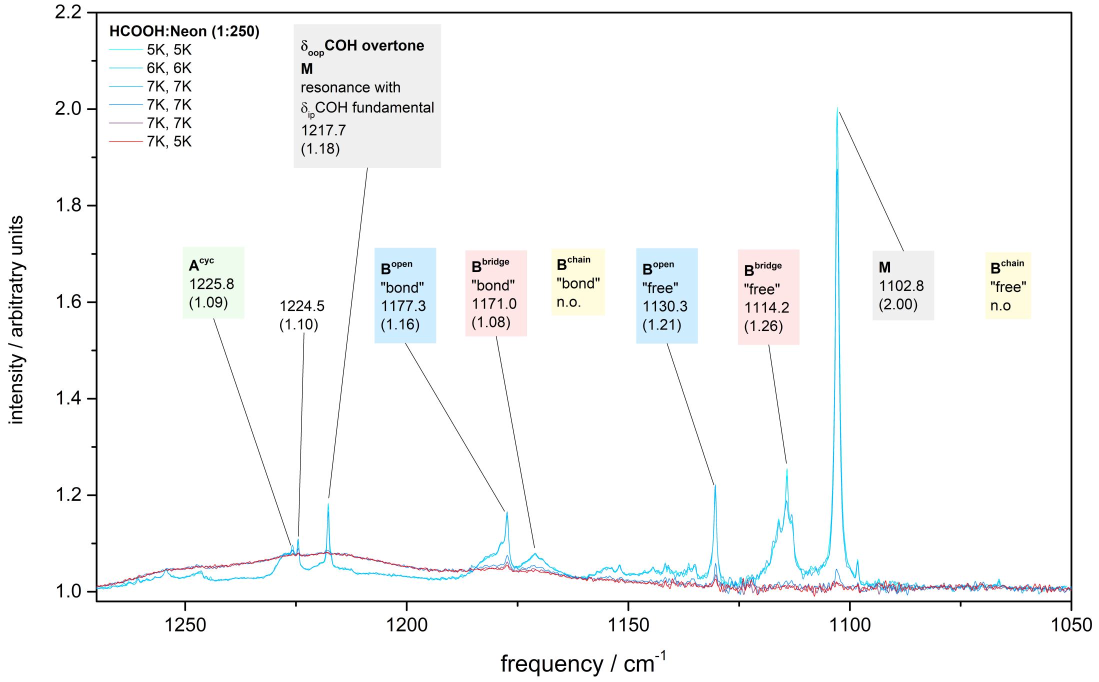

1.8 δ_{oop}CH region [1100–1000 cm^{-1}]

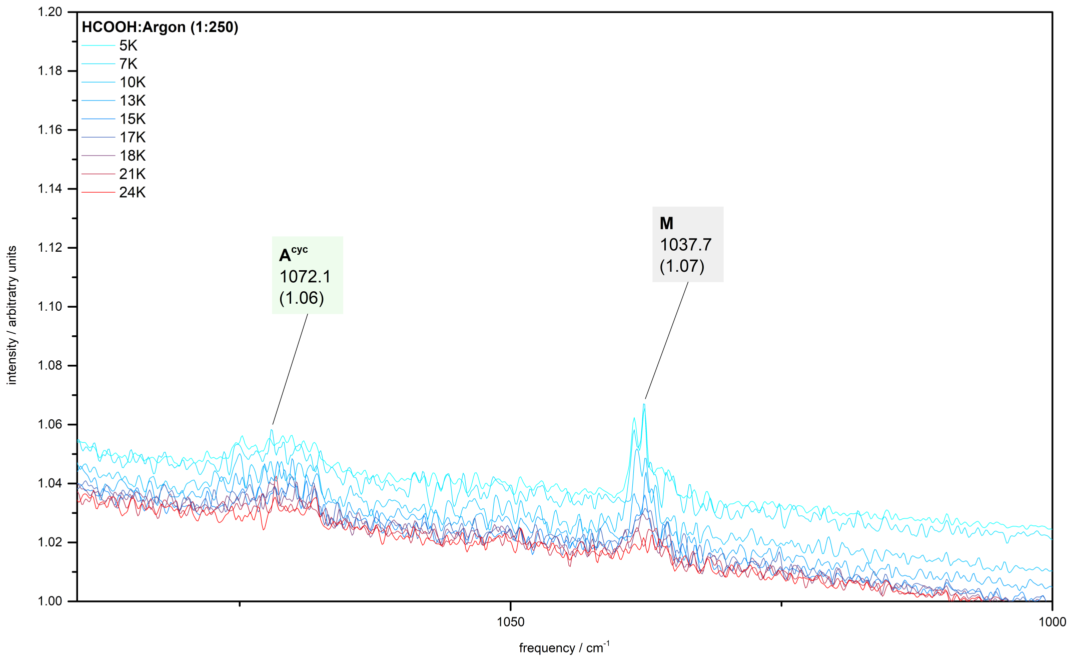

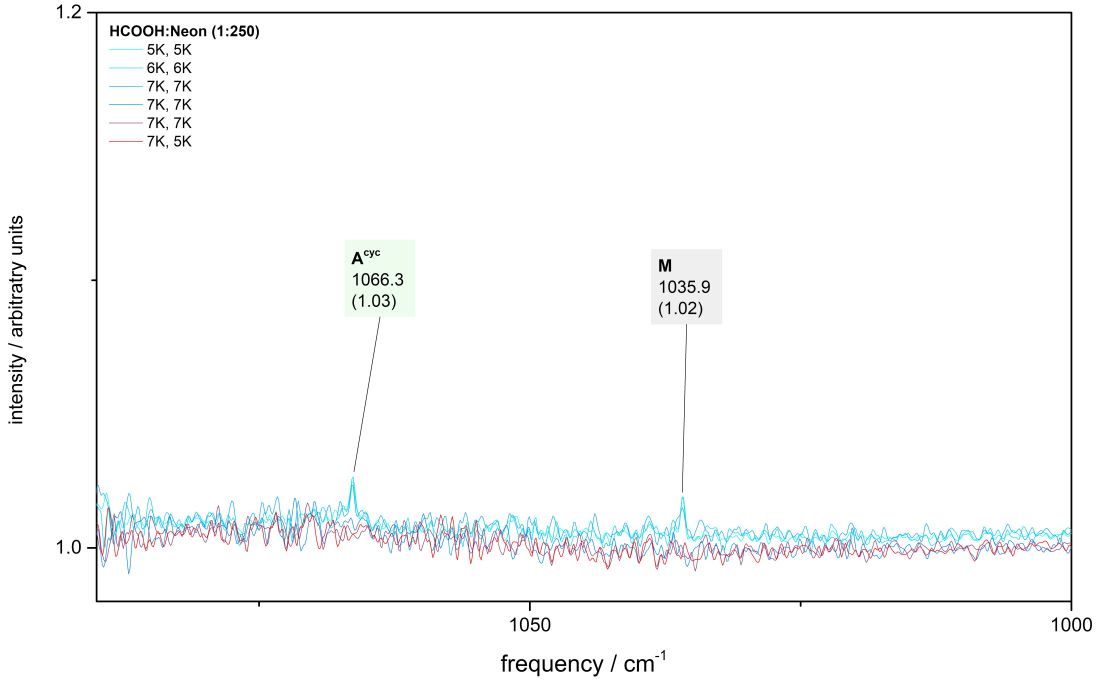

1.9 δ_{oop}COH region [1000–800 cm^{-1}]

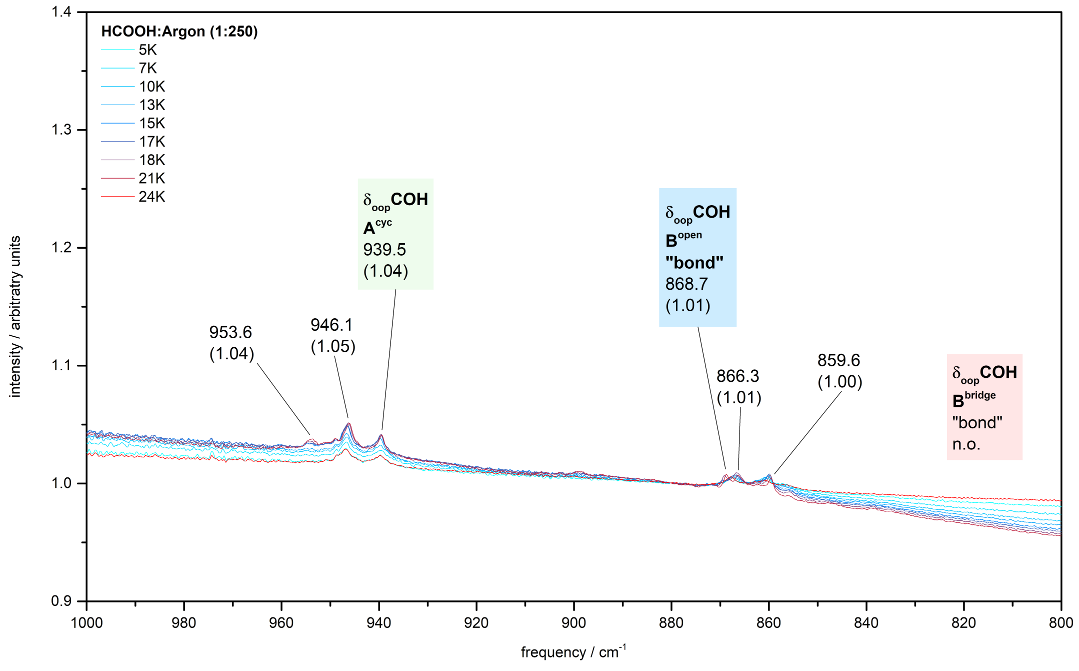

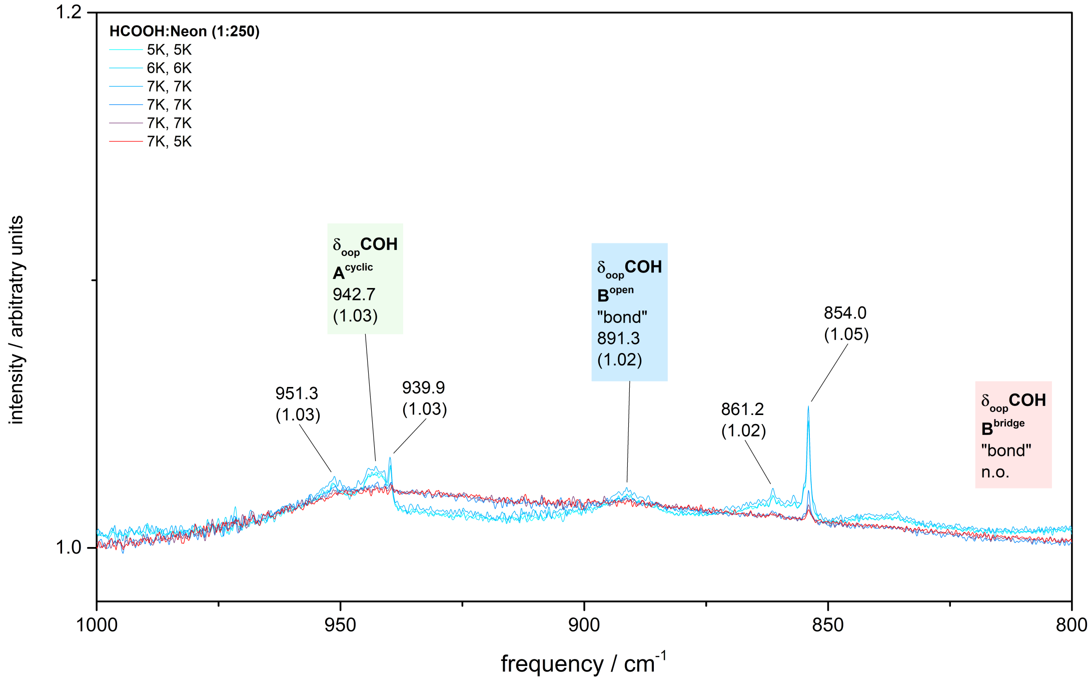

1.10 δ_{oop}COH & δ_{ip}OCO region [800–600 cm^{-1}]

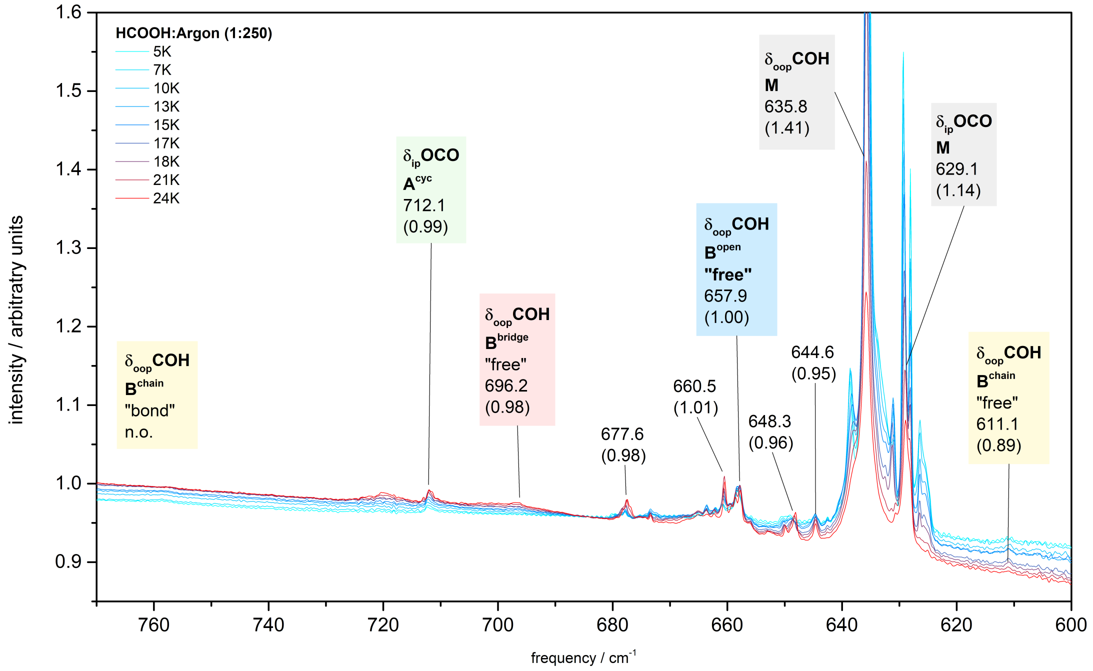

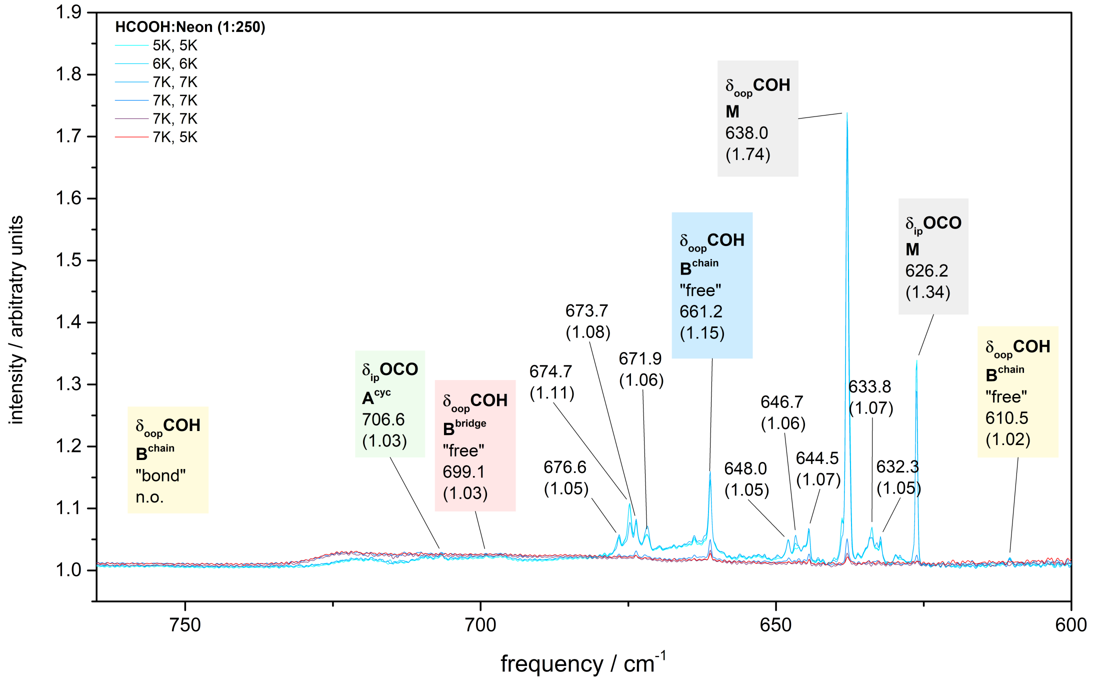

1.11 Difference spectra in argon

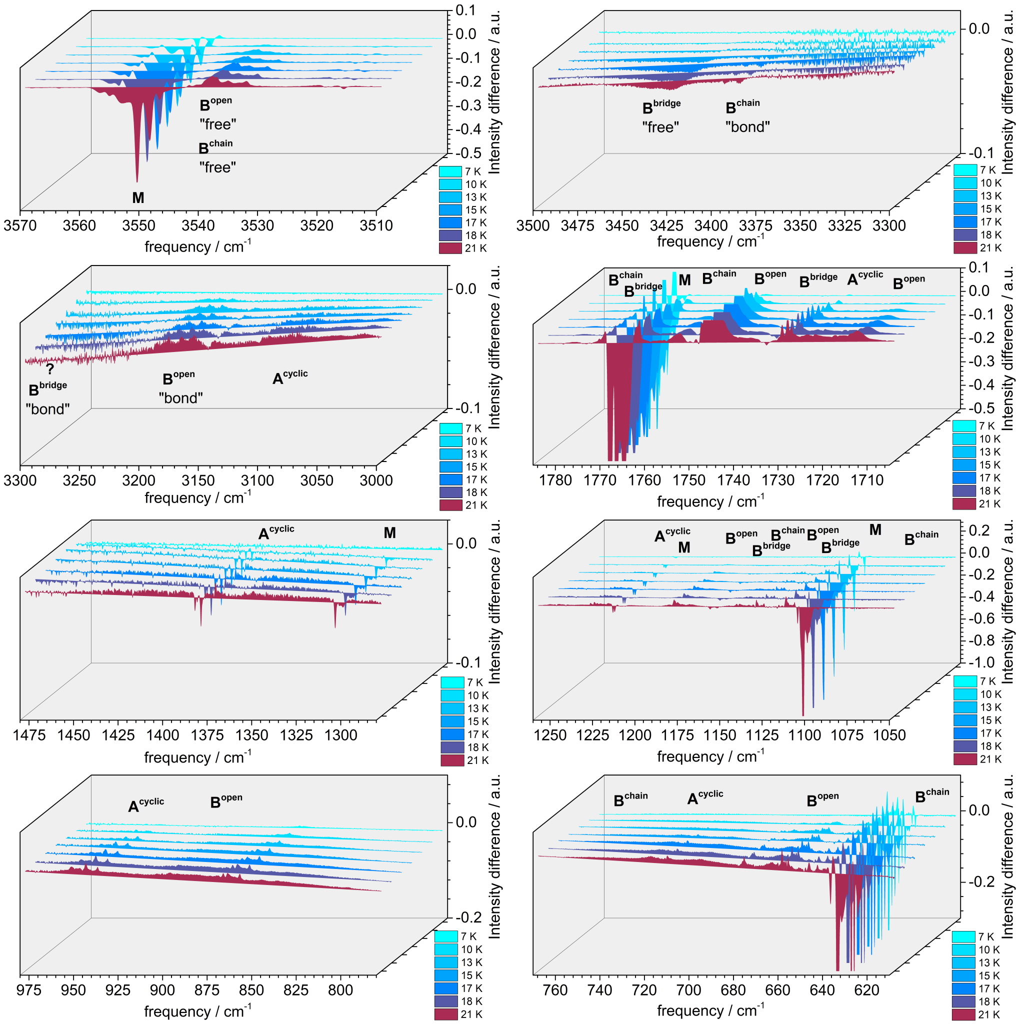

2 Computational Thermochemistry of Dimer-formation

In Table S1 we report the computed thermochemical data for the dimer formation across the 17 conformers obtained from the conformational sampling. We distinguish only the labelled A, B, C, and D conformer families, since several of the 17 structures are structurally redundant; for example, conformers 4 and 5 both correspond to the B-chain motif, while conformers 6, 7, 11, and 12 belong to the C motif. Thermochemical quantities were obtained from harmonic frequency calculations at the *wB97X-3c* level of theory at 298.15 K and 1 atm, within the ideal-gas, rigid-rotor, harmonic-oscillator (RRHO) approximation as implemented in ORCA. Vibrational entropies were evaluated using Grimme's quasi-RRHO (QRRHO) approach with a 100 cm^{-1} switching frequency to account for low-frequency vibrational modes.

Table S1: Dimer-formation thermochemistry[1] for the reaction from two *trans*-formic acid monomers to its cyclic hydrogen-bonded dimer. All values given in kcal/mol.

Conf.	Label	ΔE	ΔE+ZPE	ΔU	ΔH	ΔG
1	A-cyclic	0.0	0.0	0.0	0.0	0.0
2	B-Open	6.8	6.4	6.9	6.9	5.3
3	B-Bridge	9.6	9.0	9.5	9.5	7.4
5	B-Chain	11.0	10.2	10.9	10.9	8.6
4		10.3	9.9	10.4	10.4	8.8
7	C	12.5	11.5	12.5	12.5	9.6
6		11.6	11.0	10.9	10.9	10.3
11		13.4	12.5	13.4	13.4	10.5
12		13.4	12.5	13.4	13.4	10.5
13	C	13.8	12.6	13.6	13.6	10.5
14		13.8	12.6	13.6	13.6	10.5
10		12.9	12.1	12.8	12.8	10.8
16		14.8	13.5	14.1	14.1	10.8
17	D	14.9	13.6	14.8	14.8	11.2

[1] Calculated using the *wB97X-3c* composite method with its inherent double-ζ basis set. No empirical scaling factors were applied to the computed harmonic vibrational frequencies. Reported quantities are the relative electronic energy (ΔE), relative electronic energy including the zero-point vibrational energy (ΔE + ZPE), relative inner energy (ΔU), relative enthalpy (ΔH), and relative Gibbs free energy (ΔG)

3 Dimer-formation Frequency Shifts (A-cyclic) using Different Computational Methods

In Table S2 we show additional computational methods for obtaining the dimer-formation frequency shifts.

Table S2: Dimer-formation frequency shifts from the *trans*-formic acid monomer (M) to its cyclic hydrogen-bonded dimer ($\mathbf{A}^{\text{cyclic}}$), as determined by various theoretical calculations and compared to gas phase experiment.

**Label**		**Exp.**[1]	**Anharmonic**[2]		**Harmonic**[2]		**Harmonic (DFT)**[2]					
			**VCI**	**VPT2**	**CCSD(T)**	**MP2**	**BP86**	**PBE**	**M06**	**B3LYP**	**revDSD**	**ωB97X-3c**
$\mathbf{A}^{\text{cyclic}}$	ν**OH**	-487	-541	-473	-451	-490	-592	-599	-418	-575	-466	-563
$\mathbf{A}^{\text{cyclic}}$	ν**C−H**	-3	15	-36	8	8	-224	-201	9	3	10	6
$\mathbf{A}^{\text{cyclic}}$	ν**C=O**	-31	-36	-38	-32	-24	-55	-55	-42	-47	-31	-42
$\mathbf{A}^{\text{cyclic}}$	δ_{ip}**CH**	28	25	26	46	41	79	79	33	46	40	38
$\mathbf{A}^{\text{cyclic}}$	δ_{ip}**COH**	66	60	60	89	99	102	98	87	103	93	102
$\mathbf{A}^{\text{cyclic}}$	ν**C−O**	125	124	123	120	131	170	166	117	142	125	130
$\mathbf{A}^{\text{cyclic}}$	δ_{oop}**CH**	36	23	25	43	47	104	98	28	48	42	42
$\mathbf{A}^{\text{cyclic}}$	δ_{oop}**COH**	303	294	308	311	290	309	304	273	318	291	338
$\mathbf{A}^{\text{cyclic}}$	δ_{ip}**OCO**	82	82	78	82	84	118	117	78	96	82	90

[1] Gas phase reference data as compiled by Nejad[1].

[2] Anharmonic vibrational calculations in this work were carried out using vibrational configuration interaction (VCI) and, alternatively, second-order vibrational perturbation theory (VPT2) based on potential energy surface at high-level of electronic structure theory (see Part I of this series).

[3] Harmonic vibrational frequency calculations were carried out at several levels of electronic structure theory:

- CCSD(T)-F12 using the cc-pVTZ-F12 basis set
- MP2 using the def2-TZVP basis set
- B3LYP, BP86, PBE, and M06 using the def2-TZVP with the def2/J and def2/JK auxiliary basis sets and D3 (D3zero in case of M06) dispersion correction
- revDSD-PBEBP86/2021 using def2-TZVP with the def2/J and def2/JK and def2-TZVP/C auxiliary basis sets
- wB97X-3c composite method using an inherent double-zeta basis

No empirical scaling factors were applied to the computed vibrational frequencies.

4 Dimer-formation Frequency Shifts for the A-cylcic, B-open, B-Bridge, B-chain Conformers

5 Normal Mode Decomposition of Formic Acid Dimer Conformers

The following section contains the analysis of the normal mode decomposition utilized within the NOMODECO framework. For each conformer the optimal internal coordinate set is determined using both intra- and intermolecular primitive internal coordinates (ICs). The resulting contribution table allows, together with a visual inspection of the normal mode for a determination of the vibrational notation.
In order to generate complete sets of ICs additional intermolecular coordinates are needed. Here Nomodeco construct coordinates using both Acceptor-Donor and Acceptor-H distances. For the given conformers the following hydrogen bonds were detected by the software:

- **B-Open**: (H2,O3)
- **B-Bridge**: (H1,O4), (H2,O3)
- **B-Chain**: (H2, O1)

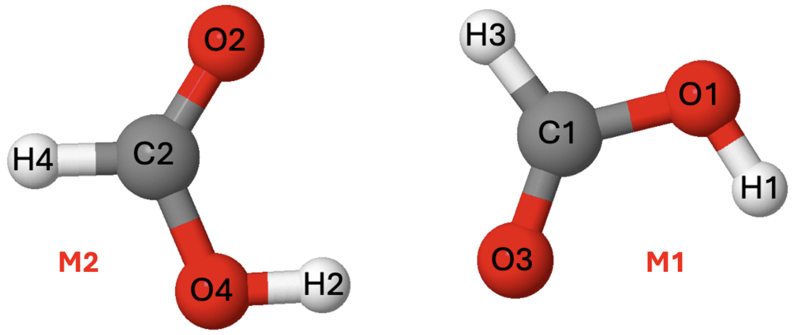

Figure S1: Labeling of Confomer **B-Open** $(HCOOH)_2$ in C_s symmetry. Monomers (**M1**) and (**M2**) have atoms {C1O1O3H1H3} an {C2O2O4H2H4} respectively.

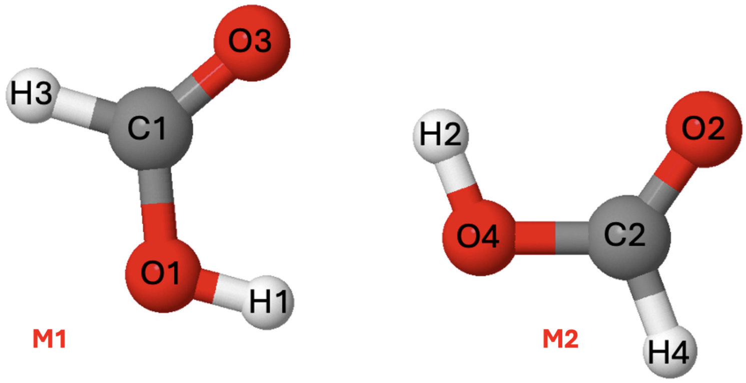

Figure S2: Conformer **B-Bridge** of $(HCOOH)_2$ in C_s symmetry. Monomers (**M1**) and (**M2**) have atoms {C1O1O3H1H3} and {C2O2O4H2H4} respectively.

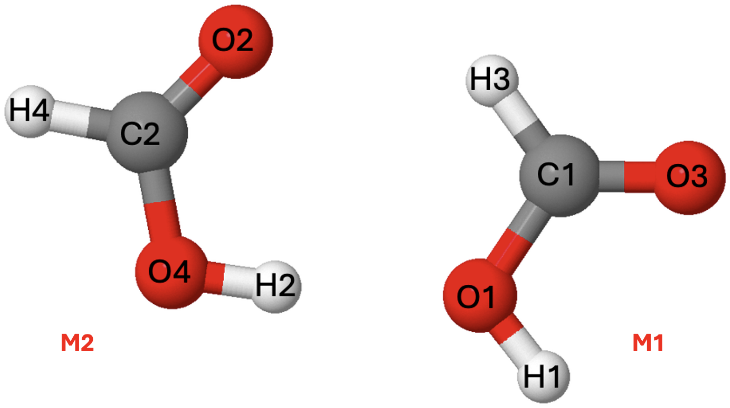

Figure S3: Conformer **B-chain** of $(HCOOH)_2$ in C_s symmetry. Monomers (**M1**) and (**M2**) have atoms {C1O1O3H1H3} and {C2O2O4H2H4} respectively.

5.1 B-Open conformer

	64	101	106	146	170	193	710	729	736	900	1205	1225	1284	1325	1440	1496	1537	1555	1950	1991	3244	3316	3901	4099
(O3, O4)	0.0	0.0	1.5	8.9	77.0	0.0	3.0	0.0	0.3	0.0	0.0	0.0	0.0	0.0	0.0	0.0	0.0	0.0	0.0	0.0	0.0	0.0	0.0	0.0
(O2, C2)	0.0	0.0	0.3	0.0	0.4	0.0	0.0	0.0	0.7	0.0	0.0	0.0	0.0	1.8	0.0	0.2	0.0	3.1	15.4	49.1	0.7	0.0	0.0	0.0
(C2, O4)	0.0	0.0	0.3	0.0	0.7	0.0	0.0	0.0	8.0	0.0	0.0	0.0	0.9	54.5	0.6	7.6	0.0	0.1	1.5	5.8	0.1	0.0	0.0	0.0
(C2, H4)	0.0	0.0	0.0	0.0	0.0	0.0	0.0	0.0	0.0	0.0	0.0	0.0	0.0	0.0	0.0	0.1	0.0	0.0	0.0	0.1	98.7	0.0	0.0	0.0
(H2, O4)	0.0	0.0	0.1	0.0	0.2	0.0	0.0	0.0	0.0	0.0	0.0	0.0	0.0	0.0	0.0	0.2	0.0	0.0	0.1	0.0	0.1	0.0	99.6	0.0
(H1, O1)	0.0	0.0	0.0	0.0	0.0	0.0	0.0	0.0	0.0	0.0	0.0	0.0	0.1	0.0	0.3	0.0	0.0	0.0	0.0	0.0	0.0	0.0	0.0	99.9
(O1, C1)	0.0	0.0	0.2	0.0	1.0	0.0	6.2	0.0	0.1	0.0	0.0	0.0	49.4	0.6	25.1	0.2	1.2	0.0	5.3	1.4	0.0	0.1	0.0	0.0
(C1, O3)	0.0	0.0	0.9	0.1	0.2	0.0	0.2	0.0	0.0	0.0	0.0	0.0	2.3	0.0	1.5	0.0	2.0	0.0	51.7	14.6	0.0	0.8	0.0	0.0
(C1, H3)	0.0	0.0	0.5	0.1	0.0	0.0	0.0	0.0	0.0	0.0	0.0	0.0	0.0	0.0	0.1	0.0	0.0	0.0	0.2	0.1	0.0	98.7	0.0	0.0
(C1, O3, O4)	0.0	0.0	70.7	53.2	1.1	0.0	5.0	0.0	0.3	0.0	0.0	0.0	0.3	0.1	0.8	0.0	0.1	0.0	0.0	0.0	0.0	0.0	0.0	0.0
(O2, C2, H4)	0.0	0.0	0.4	0.0	0.7	0.0	0.1	0.0	20.6	0.0	0.0	0.0	0.1	1.5	0.4	35.1	0.0	40.5	1.6	6.4	0.2	0.0	0.0	0.0
(O4, C2, H4)	0.0	0.0	0.2	0.5	0.0	0.0	0.1	0.0	37.0	0.0	0.0	0.0	0.1	13.9	0.0	12.6	0.0	42.5	3.6	12.4	0.1	0.0	0.1	0.0
(C2, O4, H2)	0.0	0.0	0.0	9.7	2.4	0.0	0.3	0.0	7.9	0.0	0.0	0.0	1.1	17.7	1.5	30.7	0.2	9.4	1.2	3.1	0.1	0.0	0.0	0.0
(H1, O1, C1)	0.0	0.0	0.1	0.1	0.0	0.0	19.5	0.0	0.1	0.0	0.0	0.0	25.5	0.8	51.1	0.4	1.0	0.0	2.0	0.6	0.0	0.1	0.0	0.0
(O1, C1, H3)	0.0	0.0	0.1	0.9	1.1	0.0	46.2	0.0	0.2	0.0	0.0	0.0	14.5	0.1	0.0	0.8	43.2	0.1	11.4	3.9	0.0	0.2	0.0	0.0
(O3, C1, H3)	0.0	0.0	0.1	0.1	0.0	0.0	18.9	0.0	0.0	0.0	0.0	0.0	5.4	0.1	18.1	0.2	52.0	0.1	5.8	2.0	0.0	0.1	0.0	0.0
(O3, H2, O4)	0.0	0.0	23.8	26.4	15.2	0.0	0.4	0.0	24.9	0.0	0.0	0.0	0.3	8.8	0.5	12.1	0.2	4.0	0.1	0.6	0.0	0.0	0.2	0.0
(O3, H2, O4)	2.9	0.9	0.0	0.0	0.0	21.9	0.0	0.1	0.0	72.5	1.9	0.1	0.0	0.0	0.0	0.0	0.0	0.0	0.0	0.0	0.0	0.0	0.0	0.0
(C2, H4, O2, O4)	5.2	0.2	0.0	0.0	0.0	13.7	0.0	0.0	0.0	0.1	12.6	0.0	0.0	0.0	0.0	0.0	0.0	0.0	0.0	0.0	0.0	0.0	0.0	0.0
(C1, H3, O1, O3)	0.7	54.5	0.5	0.0	0.0	0.1	0.0	0.1	0.0	0.1	0.1	96.4	0.0	0.0	0.0	0.0	0.0	0.0	0.0	0.0	0.0	0.0	0.0	0.0
(O1, C1, O3, O4)	35.7	5.5	0.1	0.0	0.0	1.8	0.0	0.6	0.0	0.0	0.0	0.8	0.0	0.0	0.0	0.0	0.0	0.0	0.0	0.0	0.0	0.0	0.0	0.0
(O2, C2, O4, O3)	29.4	0.1	0.0	0.0	0.0	52.7	0.0	0.1	0.0	1.1	22.4	0.0	0.0	0.0	0.0	0.0	0.0	0.0	0.0	0.0	0.0	0.0	0.0	0.0
(C1, O3, O4, C2)	9.3	31.4	0.3	0.0	0.0	6.0	0.0	1.0	0.0	0.2	2.0	1.5	0.0	0.0	0.0	0.0	0.0	0.0	0.0	0.0	0.0	0.0	0.0	0.0
(H2, O4, C2, H4)	3.4	0.8	0.0	0.0	0.0	3.5	0.0	0.1	0.0	26.1	61.1	0.0	0.0	0.0	0.0	0.0	0.0	0.0	0.0	0.0	0.0	0.0	0.0	0.0
(H1, O1, C1, O3)	13.5	6.6	0.1	0.0	0.0	0.3	0.0	98.0	0.0	0.1	0.0	1.2	0.0	0.0	0.0	0.0	0.0	0.0	0.0	0.0	0.0	0.0	0.0	0.0

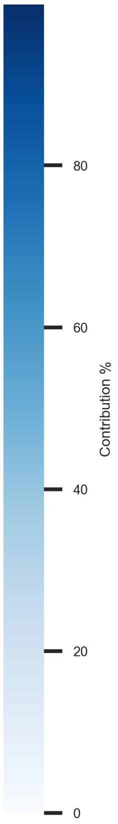

Figure S4: Contribution table for conformer **B-Open** $(HCOOH)_2$ in C_s symmetry. Rows represent internal coordinates (ICs) and columns represent the harmonic frequencies (ω in cm^{-1}).

Table S3: Vibrational notation for conformer **B-Open** $(HCOOH)_2$ in C_s symmetry, determined through the analysis of the computed intrinsic frequencies and contribution table.[1]

q_1	ω	ω^I	$\lvert\omega - \omega^I\rvert$	Label[2]	main contributing IC
1	64	342	278	ω(M1/M2) "wagging"	τ(C1C1O3O4) 35.7 % & τ(O2C2O4O3) 29.4 %
2	100.81	1165	1065	τ(M1) "twist"	γ(C1H3O1O3) 54.5 % & τ(C1O3O4C2) 31.4 %
3	106	117	11	δ_{ip}(M1/M2) "bend"	ϕ(C1O3O4) 70.7 %
4	146	117	29	δ_{ip}(M1/M2) "bend"	ϕ(C1O3O4) 53.2 %
5	170	210	40	ν(M1/M2)	r(O3O4) 77.0 %
6	193	662	469	τ(M2) "twist"	τ(O2C2O4O3) 52.7 %
7	710	1221	511	M1 δ_{ip}(COH)	ϕ(O1C1H3) 46.2 & & ϕ(H1O1C1) 19.5 %
8	729	762	33	M1 δ_{oop}(OH)	τ(H1O1C1O3) 98.0 %
9	736	1256	520	M2 δ_{ip}(COH)	ϕ(O4C2H4) 37.0 % & ϕ'(O3H2O4) 24.9 &
10	900	887	13	M2 δ_{oop}(OH)	ϕ'(O3H2O4) 72.5 &
11	1205	812	393	M2 δ_{oop}(CH)	τ(H2O4C2H4) 61.4 %
12	1225	1165	60	M1 δ_{oop}(CH)	γ(C1H3O1O3) 96.4 %
13	1284	1322	36	M1 ν(C-O)	r(O1C1) 49.4 %
14	1325	1339	14	M2 ν(C-O)	r(C2O4) 54.5 %
15	1440	1376	64	M1 δ_{ip}(OH)	ϕ(H1O1C1) 51.1 %
16	1496	1298	198	M2 δ_{ip}(OH)	ϕ(O2C2H4) 35.1 % & ϕ(C2O4H2) 30.7 %
17	1538	1308	230	M1 δ_{ip}(CH)	ϕ(O3C1H3) 52.0 % & ϕ(O1C1H3) 43.2 %
18	1555	1256	299	M2 δ_{ip}(CH)	ϕ(O4C2H4) 42.5 % & ϕ(O2C2H4) 40.5 %
19	1950	1908	42	M1 ν(C=O)	r(C1O3) 51.7 %
20	1991	1912	79	M2 ν(C=O)	r(O2C2) 49.1 %
21	3244	3239	5	M2 ν(CH)	r(C2H4) 98.7 %
22	3316	3307	9	M1 ν(CH)	r(C1H3) 98.7 %
23	3901	3899	2	M2 ν(OH) "bridge"	r(H2O4) 99.6 %
24	4099	4100	1	M1 ν(OH) "open"	r(H1O1) 99.9 %

[1] For each normal mode, given in the computational notation q_i, the harmonic ω and intrinsic frequency ω^I are given in cm^{-1}, together with the main contributing internal coordinates (ICs).

[2] Assignment is formulated in the so-called chemist notation. Abbreviations: ν=stretch, δ_{ip}=in-plane bend, ω=wagging, ρ=rocking, τ=torsion

5.2 B-Bridge conformer

	21	70	115	138	169	203	715	735	747	847	1193	1201	1269	1304	1424	1472	1540	1550	1981	2023	3249	3267	3987	4043
(O1, O4)	0.0	18.6	90.8	0.0	0.0	2.5	0.2	1.3	0.0	0.0	0.0	0.0	0.3	0.1	0.0	0.1	0.0	0.0	0.0	0.0	0.0	0.0	0.0	0.0
(O2, C2)	0.0	0.2	0.1	0.0	0.0	0.1	0.4	0.2	0.0	0.0	0.0	0.0	1.1	0.0	1.1	0.1	0.9	0.6	1.8	70.1	0.8	0.0	0.0	0.0
(C2, O4)	0.0	0.3	0.1	0.0	0.0	1.0	5.9	2.6	0.0	0.0	0.0	0.0	58.3	7.2	11.7	0.0	0.5	0.2	0.1	5.7	0.1	0.0	0.0	0.0
(C2, H4)	0.0	0.0	0.0	0.0	0.0	0.0	0.0	0.0	0.0	0.0	0.0	0.0	0.0	0.0	0.1	0.0	0.0	0.0	0.0	0.1	98.6	0.1	0.0	0.0
(H2, O4)	0.0	0.2	0.3	0.0	0.0	0.1	0.1	0.0	0.0	0.0	0.0	0.0	0.0	0.0	0.0	0.0	0.0	0.0	0.0	0.0	0.0	0.0	94.7	5.0
(H1, O1)	0.0	0.0	0.0	0.0	0.0	0.0	0.0	0.0	0.0	0.0	0.0	0.0	0.1	0.0	0.0	0.2	0.0	0.0	0.0	0.0	0.0	0.0	5.2	94.9
(O1, C1)	0.0	0.0	0.3	0.0	0.0	0.0	1.9	6.6	0.0	0.0	0.0	0.0	3.9	57.2	2.0	11.4	0.2	0.1	6.6	0.1	0.0	0.1	0.0	0.0
(C1, O3)	0.0	0.0	0.5	0.0	0.0	0.0	0.5	0.3	0.0	0.0	0.0	0.0	0.1	1.8	0.1	0.7	0.4	2.3	63.0	1.5	0.0	0.7	0.0	0.0
(C1, H3)	0.0	0.0	0.0	0.0	0.0	0.0	0.0	0.0	0.0	0.0	0.0	0.0	0.0	0.0	0.0	0.1	0.0	0.0	0.1	0.0	0.1	98.6	0.0	0.0
(H2, O4, O1)	0.0	47.2	0.3	0.0	0.0	4.4	6.3	3.7	0.0	0.0	0.0	0.0	4.2	0.0	7.9	0.5	0.1	0.2	0.0	0.1	0.0	0.0	0.0	0.0
(O4, O1, C1)	0.0	30.4	4.7	0.0	0.0	79.5	2.5	12.1	0.0	0.0	0.0	0.0	0.1	0.0	0.0	0.4	0.0	0.0	0.7	0.0	0.0	0.1	0.0	0.0
(O2, C2, H4)	0.0	0.1	0.2	0.0	0.0	1.1	15.2	6.1	0.0	0.0	0.0	0.0	0.3	0.0	21.7	3.9	34.0	14.0	0.1	6.2	0.2	0.0	0.0	0.0
(O4, C2, H4)	0.0	0.5	0.6	0.0	0.0	0.8	28.6	11.8	0.0	0.0	0.0	0.0	7.9	1.4	1.4	2.8	30.9	13.3	0.2	13.3	0.1	0.0	0.1	0.0
(C2, O4, H2)	0.0	2.0	0.1	0.0	0.0	8.2	8.3	3.4	0.0	0.0	0.0	0.0	15.3	0.1	50.7	2.3	0.6	1.1	0.0	2.1	0.1	0.0	0.0	0.0
(H1, O1, C1)	0.0	0.3	1.0	0.0	0.0	2.0	7.6	4.7	0.0	0.0	0.0	0.0	6.5	16.6	2.4	34.0	0.4	3.5	2.9	0.1	0.0	0.1	0.0	0.0
(O1, C1, H3)	0.0	0.0	0.0	0.0	0.0	0.1	14.4	31.8	0.0	0.0	0.0	0.0	1.2	14.0	0.9	8.3	15.2	32.2	16.4	0.4	0.0	0.1	0.0	0.1
(O3, C1, H3)	0.0	0.0	1.0	0.0	0.0	0.2	8.1	15.4	0.0	0.0	0.0	0.0	0.8	1.6	0.0	35.2	16.7	32.5	8.0	0.2	0.0	0.2	0.0	0.0
(C2, O2, O4, H4)	2.9	0.0	0.0	6.3	27.2	0.0	0.0	0.0	0.3	1.1	77.2	0.4	0.0	0.0	0.0	0.0	0.0	0.0	0.0	0.0	0.0	0.0	0.0	0.0
(C1, O1, O3, H3)	0.1	0.0	0.0	4.3	6.8	0.0	0.0	0.0	0.2	0.1	0.0	2.8	0.0	0.0	0.0	0.0	0.0	0.0	0.0	0.0	0.0	0.0	0.0	0.0
(O1, O4, C2, O2)	75.3	0.0	0.0	22.1	0.2	0.0	0.0	0.0	0.3	0.0	13.4	0.4	0.0	0.0	0.0	0.0	0.0	0.0	0.0	0.0	0.0	0.0	0.0	0.0
(H3, C1, O1, O4)	0.1	0.0	0.0	9.0	47.1	0.0	0.0	0.0	0.2	0.0	0.0	83.7	0.0	0.0	0.0	0.0	0.0	0.0	0.0	0.0	0.0	0.0	0.0	0.0
(C1, O1, O4, H2)	0.2	0.0	0.0	19.5	5.5	0.0	0.0	0.0	15.8	33.2	0.3	4.9	0.0	0.0	0.0	0.0	0.0	0.0	0.0	0.0	0.0	0.0	0.0	0.0
(C2, O4, O1, H1)	17.1	0.0	0.0	38.2	12.4	0.0	0.0	0.0	29.6	2.4	6.6	1.8	0.0	0.0	0.0	0.0	0.0	0.0	0.0	0.0	0.0	0.0	0.0	0.0
(H2, O4, C2, O2)	3.7	0.0	0.0	0.0	0.0	0.0	0.0	0.0	14.5	47.4	2.4	0.0	0.0	0.0	0.0	0.0	0.0	0.0	0.0	0.0	0.0	0.0	0.0	0.0
(H1, O1, C1, O3)	0.5	0.0	0.0	0.6	0.7	0.0	0.0	0.0	39.1	15.8	0.0	6.0	0.0	0.0	0.0	0.0	0.0	0.0	0.0	0.0	0.0	0.0	0.0	0.0

Figure S5: Contribution table for conformer **B-Bridge** $(HCOOH)_2$ in C_s symmetry. Rows represent internal coordinates (ICs) and columns represent the harmonic frequencies (ω in cm^{-1}).

Table S4: Vibrational notation for conformer **B-bridge** $(HCOOH)_2$ in C_s symmetry, determined through the analysis of the computed intrinsic frequencies and contribution table.[1]

q_1	ω	ω^I	$\|\omega - \omega^I\|$	Label[2]	main contributing IC
1	21	316	295	ω(M1/M2) "wagging"	τ(O1O4C2O2) 75.3 %
2	70	502	432	δ_{ip}(M1/M2) "bend"	ϕ(H2O4O1) 47.2 % & ϕ(O4O1C1) 30.4 %
3	115	160	45	ν(M1/M2)	r(O1O4) 90.8 %
4	138	562	424	τ(M1/M2) "twist"	τ(C2O4O1H1) 38.2 % & τ(O1O4C2O2) 22.1 %
5	169	1182	1013	τ(M1/M2) "twist"	τ(H3C1O1O4) 47.5 % γ(C2O2O4H3) 27.2 %
6	203	150	53	δ_{ip}(M1/M2) "bend"	ϕ(O4O1C1) 79.5 %
7	715	1249	534	M2 δ_{ip}(COH)	ϕ(O4C2H4) 28.6 % & ϕ(O2C2H3) 15.2 %
8	735	1245	510	M1 δ_{ip}(COH)	ϕ(O1C1H3) 31.8 % & ϕ(O3C1H3) 15.4 %
9	747	964	217	M1/M2 δ_{oop}(OH)	τ(H1O1C1O3) 39.1 % & τ(C2O4O1H1) 29.6 %
10	847	433	414	M1/M2 δ_{oop}(OH)	τ(H2O4C2O2) 47.4 % & τ(C1O1O4H2) 33.2 %
11	1193	1140	53	M2 δ_{oop}(CH)	γ(C2O2O4H4) 77.2 %
12	1201	1182	19	M1 δ_{oop}(CH)	τ(H3C1O1O4) 83.7 %
13	1269	1274	5	M2 ν(C-O)	r(C2O4) 58.3 %
14	1304	1321	17	M1 ν(C-O)	r(O1C1) 57.2 %
15	1424	1306	118	M2 δ_{ip}(OH)	ϕ(C2O4H2) 50.7 % & ϕ(O2C2H4) 21.7 %
16	1472	1308	164	M1 δ_{ip}(OH)	ϕ(O3C1H3) 35.2 % & ϕ(H1O1C1) 34.0 %
17	1540	1288	252	M2 δ_{ip}(CH)	ϕ(O2C2H4) 34.0 % & ϕ(O4C2H4) 30.9 %
18	1550	1308	242	M1 δ_{ip}(CH)	ϕ(O3C1H3) 32.5 % & ϕ(O1C1H3) 32.2 %
19	1981	1914	67	M1 ν(C=O)	r(C1O3) 63.0 %
20	2023	1971	52	M2 ν(C=O)	r(O2C2) 70.1 %
21	3249	3242	7	M2 ν(CH)	r(C2H4) 98.6 %
22	3267	3261	6	M1 ν(CH)	r(C1H3) 98.6 %
23	3987	3990	3	M2 ν(OH) "bridge"	r(H2O4) 94.7 %
24	4043	4040	3	M1 ν(OH) "bridge"	r(H1O1) 94.9 %

[1] For each normal mode, given in the spectroscopic notation ν together with the irreducible representation in brackets, , the harmonic ω and intrinsic frequency ω^I are given in cm^{-1}, together with the main contributing internal coordinates (ICs).

[2] Assignment is formulated in the so-called chemist notation. Abbreviations: ν=stretch, δ_{ip}=in-plane bend, ω=wagging, ρ=rocking, τ=torsion

5.3 B-Chain conformer

	43	91	95	135	138	155	689	696	729	816	1201	1207	1232	1307	1402	1481	1537	1553	1992	2019	3254	3307	4012	4098
(H2, O1)	0.0	0.0	1.4	33.4	44.1	0.0	0.0	1.9	0.4	0.0	0.0	0.0	0.1	0.2	0.0	0.0	0.0	0.0	0.0	0.0	0.0	0.0	1.9	0.0
(O2, C2)	0.0	0.0	0.3	0.1	0.1	0.0	0.0	0.1	0.5	0.0	0.0	0.0	0.0	1.6	0.1	0.3	0.2	2.5	66.0	0.1	0.7	0.0	0.0	0.0
(C2, O4)	0.0	0.0	0.4	0.1	0.2	0.0	0.0	0.3	6.4	0.0	0.0	0.0	3.1	51.6	0.6	8.1	0.1	0.1	6.7	0.0	0.1	0.0	0.0	0.0
(C2, H4)	0.0	0.0	0.0	0.0	0.0	0.0	0.0	0.0	0.0	0.0	0.0	0.0	0.0	0.0	0.0	0.1	0.0	0.0	0.1	0.0	98.7	0.0	0.0	0.0
(H2, O4)	0.0	0.0	0.0	0.0	0.0	0.0	0.0	0.0	0.0	0.0	0.0	0.0	0.0	0.0	0.1	0.1	0.0	0.0	0.0	0.0	0.1	0.0	97.6	0.3
(H1, O1)	0.0	0.0	0.0	0.0	0.0	0.0	0.0	0.0	0.0	0.0	0.0	0.0	0.0	0.0	0.2	0.0	0.0	0.0	0.0	0.0	0.0	0.0	0.3	99.6
(O1, C1)	0.0	0.0	1.2	0.0	0.1	0.0	0.0	8.3	0.7	0.0	0.0	0.0	73.3	2.3	6.7	0.1	0.2	0.1	0.0	4.5	0.0	0.1	0.0	0.0
(C1, O3)	0.0	0.0	0.0	0.0	0.2	0.0	0.0	0.1	0.0	0.0	0.0	0.0	0.6	0.0	1.6	0.0	1.4	0.5	0.1	71.6	0.0	1.0	0.0	0.0
(C1, H3)	0.0	0.0	0.8	0.0	0.0	0.0	0.0	0.0	0.0	0.0	0.0	0.0	0.0	0.0	0.1	0.0	0.0	0.0	0.0	0.3	0.0	98.5	0.0	0.0
(H2, O1, C1)	0.0	0.0	15.7	59.6	11.8	0.0	0.0	0.8	5.4	0.0	0.0	0.0	0.1	0.8	0.6	1.5	0.0	0.2	0.1	0.2	0.0	0.0	0.0	0.0
(O2, C2, H4)	0.0	0.0	0.4	0.0	0.3	0.0	0.0	1.1	17.1	0.0	0.0	0.0	0.0	1.4	1.0	27.4	12.5	32.5	7.4	0.0	0.2	0.0	0.0	0.0
(O4, C2, H4)	0.0	0.0	0.2	0.3	0.1	0.0	0.0	2.3	32.2	0.0	0.0	0.0	0.2	12.4	0.1	7.2	11.6	33.1	15.3	0.0	0.1	0.0	0.1	0.0
(C2, O4, H2)	0.0	0.0	0.0	2.7	3.6	0.0	0.0	1.9	8.5	0.0	0.0	0.0	2.2	17.8	1.4	34.0	0.4	6.0	3.7	0.0	0.1	0.0	0.0	0.0
(H1, O1, C1)	0.0	0.0	0.0	0.4	0.6	0.0	0.0	18.5	0.3	0.0	0.0	0.0	13.7	0.2	47.4	0.6	1.9	0.8	0.0	2.5	0.0	0.1	0.0	0.0
(O1, C1, H3)	0.0	0.0	0.1	0.1	0.0	0.0	0.0	42.4	1.7	0.0	0.0	0.0	6.0	0.8	6.9	2.0	36.0	10.4	0.0	14.5	0.0	0.1	0.0	0.1
(O3, C1, H3)	0.0	0.0	0.4	1.0	0.0	0.0	0.0	18.1	0.7	0.0	0.0	0.0	0.2	0.1	32.4	3.0	35.5	10.5	0.0	6.3	0.0	0.2	0.0	0.0
(O4, H2, O1)	0.0	0.0	79.1	2.2	38.9	0.0	0.0	4.2	26.1	0.0	0.0	0.0	0.4	10.8	0.7	15.7	0.2	3.1	0.6	0.0	0.0	0.0	0.0	0.0
(O4, H2, O1)	0.3	0.7	0.0	0.0	0.0	40.0	1.2	0.0	0.0	38.0	0.5	0.0	0.0	0.0	0.0	0.0	0.0	0.0	0.0	0.0	0.0	0.0	0.0	0.0
(C2, H4, O2, O4)	3.1	0.3	0.0	0.0	0.0	31.2	0.0	0.0	0.0	0.0	91.7	2.1	0.0	0.0	0.0	0.0	0.0	0.0	0.0	0.0	0.0	0.0	0.0	0.0
(C1, H3, O1, O3)	8.3	66.8	0.0	0.0	0.0	3.6	6.9	0.0	0.0	0.0	0.8	84.3	0.0	0.0	0.0	0.0	0.0	0.0	0.0	0.0	0.0	0.0	0.0	0.0
(O3, C1, O1, O4)	62.9	10.0	0.0	0.0	0.0	8.4	0.3	0.0	0.0	0.0	0.0	7.2	0.0	0.0	0.0	0.0	0.0	0.0	0.0	0.0	0.0	0.0	0.0	0.0
(C1, O1, O4, C2)	17.1	19.8	0.0	0.0	0.0	16.3	4.8	0.0	0.0	0.3	3.5	4.1	0.0	0.0	0.0	0.0	0.0	0.0	0.0	0.0	0.0	0.0	0.0	0.0
(H2, O4, C2, O2)	3.4	0.5	0.0	0.0	0.0	0.4	3.4	0.0	0.0	61.4	3.4	0.1	0.0	0.0	0.0	0.0	0.0	0.0	0.0	0.0	0.0	0.0	0.0	0.0
(H1, O1, C1, O3)	4.9	1.8	0.0	0.0	0.0	0.2	83.4	0.0	0.0	0.3	0.0	2.3	0.0	0.0	0.0	0.0	0.0	0.0	0.0	0.0	0.0	0.0	0.0	0.0

Figure S6: Contribution table for conformer **B-Chain** $(HCOOH)_2$ in C_s symmetry. Rows represent internal coordinates (ICs) and columns represent the harmonic frequencies (ω in cm^{-1}).

Table S5: Vibrational notation for conformer **B-chain** $(HCOOH)_2$ in C_s symmetry, determined through the analysis of the computed intrinsic frequencies and contribution table.[1]

q_1	ω	ω^I	$\lvert\omega$ - $\omega^I\rvert$	Label[2]	main contributing IC
1	43	510	467	ω(M1/M2) "wagging"	τ(O3C1O1O4) 62.9 %
2	91	984	893	τ(M1) "twist"	γ(C1H3O1O3) 66.8 %
3	95	416	321	δ_{ip}(M1/M2) "bend"	ϕ'(O4H2O1) 79.1 % & ϕ(H2O1C1) 15.7 %
4	135	294	162	δ_{ip}(M1/M2) "bend"	ϕ(H2O1C1) 59.6 % & r(H2O1) 33.4 %
5	138	67	71	ν(M1/M2)	r(H2O1) 44.1 %
6	155	428	273	τ(M2) "twist"	ϕ'(O4H2O1) 40.0 % & γ(C2H4O2O4) 31.2 %
7	689	751	62	M1 δ_{oop}(OH)	τ(H1O1C1O3) 83.4 %
8	696	1259	563	M1 δ_{ip}(COH)	ϕ(O1C1H3) 42.4 % & ϕ(H1O1C1) 18.5 %
9	729	1250	521	M2 δ_{ip}(COH)	ϕ(O4C2H4) 32.2 % & ϕ'(O4H2O1) 26.1 %
10	816	888	72	M2 δ_{oop}(OH)	τ(H2O4C2O2) 61.4 %
11	1201	1098	103	M2 δ_{oop}(CH)	γ(C2H4O2O4) 91.7 %
12	1207	984	223	M1 δ_{oop}(CH)	γ(C1H3O1O3) 84.34 %
13	1232	1219	13	M1 ν(C-O)	r(O1C1) 73.3 %
14	1307	1320	13	M2 ν(C-O)	r(C2O4) 51.6 %
15	1402	1379	23	M1 δ_{ip}(OH)	ϕ(H1O1C1) 47.4 % & ϕ(O3C1H3) 32.4 %
16	1481	1368	113	M2 δ_{ip}(OH)	ϕ(C2O4H2) 34.0 % & ϕ(O2C2H4) 27.4 %
17	1537	1259	278	M1 δ_{ip}(CH)	ϕ(O1C1H3) 36.0 % & ϕ(O3C1H3) 35.5 %
18	1553	1250	303	M2 δ_{ip}(CH)	ϕ(O4C2H4) 33.1 % & ϕ(O2C2H4) 32.5 %
19	1992	1928	64	M2 ν(C=O)	r(O2C2) 66.0 %
20	2019	1977	42	M1 ν(C=O)	r(C1O3) 71.6 %
21	3254	3248	6	M2 ν(CH)	r(C2H4) 98.7 %
22	3307	3296	11	M1 ν(CH)	r(C1H3) 98.5 %
23	4012	4016	4	M2 ν(OH) "bridge"	r(H2O4) 97.6 %
24	4098	4099	1	M1 ν(OH) "open"	r(H1O1) 99.6 %

[1] For each normal mode, given in the computational notation q_i, the harmonic ω and intrinsic frequency ω^I are given in cm^{-1}, together with the main contributing internal coordinates (ICs).

[2] Assignment is formulated in the so-called chemist notation. Abbreviations: ν=stretch, δ_{ip}=in-plane bend, ω=wagging, ρ=rocking, τ=torsion

6 Supplementary References

Notes and references

[1] A. Nejad, *PhD thesis*, Georg-August-University Göttingen, 2022.
